# Text-Based Depression Estimation Using Machine Learning With Standard Labels: Systematic Review and Meta-Analysis

**DOI:** 10.2196/82686

**Published:** 2026-02-11

**Authors:** Shengming Zhang, Chaohai Zhang, Jiaxin Zhang

**Affiliations:** 1 School of Automation and Intelligent Manufacturing Southern University of Science and Technology Shenzhen, Guangdong China; 2 Guangdong Provincial Key Laboratory of Fully Actuated System Control Theory and Technology School of Automation and Intelligent Manufacturing Southern University of Science and Technology Shenzhen, Guangdong China

**Keywords:** depression, natural language processing, standard labels, text, TRIPOD, Transparent Reporting of a multivariable prediction model for Individual Prognosis Or Diagnosis

## Abstract

**Background:**

Depression affects people’s daily lives and even leads to suicidal behavior. Text-based depression estimation using natural language processing has emerged as a feasible approach for early mental health screening. However, most existing reviews often included studies with weak depression labels, which affected the reliability of the results and further limited the practical application of the automatic depression estimation models.

**Objective:**

This review aimed to evaluate the predictive performance of text-based depression models that used standard labels, and to identify text resources, text representation, model architecture, annotation source, and reporting quality contributing to performance heterogeneity.

**Methods:**

Following PRISMA (Preferred Reporting Items for Systematic Reviews and Meta-Analyses) 2020 guidelines, we systematically searched 4 main databases (PubMed, Scopus, IEEE Xplore, and Web of Science) for studies published between 2014 and 2025. The eligible studies were included: machine learning models were developed based on the text generated by the participants and used validated scales or clinical diagnoses as depression labels. Pooled effect sizes (*r*) were calculated using random-effects meta-analysis with Hartung-Knapp-Sidik-Jonkman correction, and subgroup and meta-regression analyses were conducted to explore potential moderators.

**Results:**

We scanned 3067 articles and finally filtered 15 models from 11 studies for the meta-analysis. The overall pooled effect size was 0.605 (95% CI 0.498-0.693), indicating a large strength of association. Subgroup analyses showed that models using embedding-based text representations achieved higher performance than those using traditional features (*r*=0.741, 95% CI 0.648-0.812 vs *r*=0.514, 95% CI 0.385-0.623; *P*<.001 for subgroup difference), and deep learning architectures outperformed shallow models (*r*=0.731, 95% CI 0.660-0.789 vs *r*=0.486, 95% CI 0.352-0.599; *P*<.001). Models trained with clinician diagnoses also outperformed better than those relying on self-report scales (*r*=0.688, 95% CI 0.554-0.787 vs *r*=0.500, 95% CI 0.340-0.631; *P*=.03). Reporting quality was positively associated with model performance (β=0.085, 95% CI 0.050-0.119; *P*<.001). Begg–Mazumdar and Egger tests provided no evidence of small-study effects. Begg–Mazumdar test (Kendall τ=0.17143, *P*=.37) and the Egger test (t_14_=1.13401, 2-tailed *P*=.28) indicated no evidence of small-study effects.

**Conclusions:**

Text-based depression estimation models trained with standard depression labels demonstrate solid predictive performance, with embedding features, deep model architectures, and clinician diagnosis labels showing significantly higher performance. Transparent reporting is also positively associated with model performance. This study highlights the importance of standard labels, feature representation, and reporting quality for improving model reliability. Unlike prior reviews that included weak or heterogeneous depression labels, this study offers more clinically reliable and comparable evidence. Moreover, this review provides clearer methodological guidance for developing more consistent and practically informative text-based depression screening models.

**Trial Registration:**

PROSPERO CRD420251056902; https://www.crd.york.ac.uk/PROSPERO/view/CRD420251056902

## Introduction

### Background

Depression, a type of mental disorder, is clinically characterized by persistent and significant low mood [1]. Individuals with severe depression often experience impaired social functioning and may even exhibit suicidal behaviors [2,3]. Currently, the diagnosis of depression relies on face-to-face consultation by psychiatrists and refers to the patients’ self-reports. Although the clinical diagnosis by psychiatrists is regarded as the gold standard of depression detection [4], due to the time-constrained consultations and subjective information in psychiatry [5], the lack of clinical resources has prevented the gold standard from being widely used. To alleviate clinical burden, standardized self-reported scales have been developed based on psychiatrists’ clinical experience [6], and several of them have been demonstrated to have comparable performance to clinical diagnosis [7,8]. However, these tools partially address the shortage of clinical resources; limitations such as subjective bias and time-consuming assessments remain [9-11]. With the development of artificial intelligence technologies, the limitations of traditional depression diagnosis methods facilitate the emergence of automatic depression estimation (ADE) models based on various multimodal data sources [12,13].

Among these multimodal data, text-based features have become a popular target in depression estimation studies [14-17]. Unlike other features used in ADE models [18-20], text (language) serves as a natural medium of human communication, conveying not only semantic content but also affective states [21,22]. The ability to capture emotional cues from text aligns closely with the depression diagnostic and may provide references for estimation [23]. For example, Cariola et al [24] found that mothers with depression used more first-person singular pronouns and present-focused words in mother-child dialogues, possibly reflecting heightened self-focus and introspection. Another study demonstrated that the ratio of negative to positive language played a vital role in establishing emotional tone [25]. Additionally, text data can be sourced from various contexts, including social media posts [26,27], SMS messages [28], chat logs [17], clinical transcripts [29], and even electronic health records [30]. Wang et al [27] achieved 91% classification accuracy based on social media posts, while Liu et al [28] reported an area under the curve of 76% on SMS messages. These studies achieved notable performance and supported the feasibility of text-based ADEs. However, differences in the reported contents (eg, text sources, model construction and validation, and depression labels) across studies may lead to substantial variability in model performance, thereby affecting the reliability of the results and the further practical application.

The quality of training data is critical to model performance, and the difference in text sources affects the performance of the ADE models [31]. The datasets of existing studies could be broadly categorized into two types: (1) public datasets, such as the Distress Analysis Interview Corpus – Wizard-of-Oz (DAIC-WOZ) [32], the Audio/Visual Emotion Challenge 2014 dataset [33], the Extended Audio-Transcript Depression Corpus [34], and the Chinese Multimodal Depression Corpus [35], which are collected under standardized protocols and provide a unified benchmark for comparing algorithm performance. For example, text-only models trained on DAIC-WOZ have shown gradual improvements in recent years [36,37]. The others belong to (2) self-constructed datasets, which are more diverse compared to public datasets in collection, and are usually used for exploring model feasibility in specific populations or contexts rather than comparing model algorithms. They include transcripts of clinical interviews or therapeutic dialogues, personal essays, diaries, questionnaires, etc. (see the previous paragraph for details). These datasets tend to better reflect natural language use in real-world contexts, which enhances the practical application ability of the ADE models [38]. For example, Cheng et al [39] analyzed social media (Weibo [Sina Corporation]) texts to predict depression and found that linguistic and cultural factors can affect the model performance. While Dogrucu et al [40] used text data via smartphones for developing Moodable, an Android-based depression sensing application developed at Worcester Polytechnic Institute, and validated this application for depression estimation. Overall, these examples illustrated the importance of self-constructed datasets, while the differences in text sources and contexts may contribute to heterogeneity in model performance.

After determining the dataset, model construction and validation are the core stages of ADE studies. Previous studies mainly relied on statistical methods to manually extract linguistic features. Rude et al [41] noted that individuals with depression frequently used negations and first-person singular pronouns, while another research examined the frequency of affective words such as “anger” or “sadness” to derive participants’ emotion [42]. However, with the rapid development of natural language processing (NLP), embedding-based representations (eg, Word2Vec [43], Global Vector [44]) and pretrained models (eg, BERT [Bidirectional Encoder Representations from Transformers] [45], GPT [46]) have enhanced the semantic modeling capacity of ADE models. Niu et al [47] applied graph attention networks to capture hierarchical contextual semantics, and some studies used BERT to encode transcribed speech into context-aware sentence embeddings [48,49]. In model architecture, traditional shallow models, such as support vector machines, random forests, and decision trees, were commonly used in previous works [50], while deep learning models have also performed well in the ADE tasks recently [51-53]. Dinkel et al [51] built a multitask model based on a bidirectional gated recurrent unit, achieving an *F*_1_-score of 0.84 on DAIC-WOZ. Martinez et al [52] further improved classification performance using RoBERTa. Furthermore, the validation strategies also vary: while single-holdout testing is commonly used when datasets are large [54-56], small-sample medical texts often use repeated bootstrapping [57] or k-fold cross-validation [30,58] to ensure robustness. Overall, differences in feature representation, model architecture, and validation strategies all contribute to potential differences in model performance and generalization.

Another potential factor affecting the model performance is the quality of depression labels. Clinical diagnosis by certified psychiatrists is widely considered the gold standard of depression [1,59]. However, clinical diagnosis is costly and subject to the experience of the psychiatrists [60]. As a substitute, many text-based ADE studies used standardized self-report depression scales. Common scales include the Patient Health Questionnaire, 8 or 9 items (PHQ-8 or 9) [61], Beck Depression Inventory-II (BDI-II) [62], Zung Self-Rating Depression Scale (SDS) [63], 21-item Depression, Anxiety, and Stress Scale (DASS-21) [64], and Center for Epidemiological Studies Depression Scale (CES-D) [65]. The ADE tasks can be equal to predicting the self-reported scores. For example, Li et al [66] achieved an *F*_1_-score of 78.3% on the DAIC-WOZ dataset using PHQ-8, and another study reported a classification accuracy of 69% using PHQ-9 labels [67]. However, structured scales demonstrate good reliability and validity, but their use is often limited by ethical constraints and annotation costs. Consequently, many studies resort to weak depression labels, such as keyword matching [68,69], sentiment analysis tools [70], or self-declared depressive status from social media [71]. These approaches facilitate large-scale data collection and exploration, but there may be deviations in the reliability and validity of practical applications. For example, keyword-based labels may ignore semantic context [72], and sentiment analysis tools may confuse sadness with clinical depression [73]. Therefore, increasing emphasis has been placed on adopting gold-standard labels for model training and validation [16,31,74]. In this study, we included only those studies that use either clinical diagnosis or the PHQ-9 scale as depression labels: Other scales, such as BDI-II and CES-D, though commonly used for screening, differ in target populations and sensitivity, which may impair cross-study comparability [75]. The potential impact of annotation source on model performance is examined in our Results section.

In recent years, several systematic reviews have investigated NLP-based depression estimation. Mao et al [31] provided a multimodal overview covering facial expression, speech, and text models, highlighting the lack of model transparency and limited practical application as critical challenges. Some reviews focus on specific methods. For example, Yao et al [16] summarized depression-related studies using social media text and found that machine learning (ML) and statistical analysis methods were more prevalent. Tahir et al [76] provided a comprehensive review of ML and deep learning approaches for ADE tasks based on social media data. Nanggala et al [77] conducted a systematic review of the performance of the transformer structure in ADE tasks and noted the competitive advantages of the text modalities. Many studies have examined the use of social media text for depression estimation, analyzing feature design and training strategies [16,78]. While these previous works provided valuable insights into this field, 2 major limitations remain: First, most reviews concentrate on specific text sources (eg, social media and public datasets), which may not generalize well to practical applications [79]. Second, annotation sources are often overlooked, and weak depression labels, such as self-declared or keyword matching, are often confused without discussing the impact of their differences from standard labels on model performance [72]. Therefore, this study uses label quality as a core inclusion criterion. We systematically assessed performance and sources of heterogeneity in text-based ADE models.

### Research Aims and Structure

This review aims to evaluate the performance of text-based depression estimation models that use standard labels and to identify the potential moderators that may account for performance differences. We specifically analyzed variations in text sources, model architectures, validation strategies, annotation standards, as well as the quality of study reporting, to examine their potential influence on depression predictive performance. The structure of this review is as follows: the Introduction section outlines the background and research aims, the Methods section presents the study selection criteria, data extraction, and meta-analysis protocol, the Results section presents the outcomes of the meta-analysis, and the Discussion and Conclusion section interprets the findings and provides implications for future research and practical applications.

## Methods

### Study Design

This systematic review followed the PRISMA (Preferred Reporting Items for Systematic Reviews and Meta-Analysis) 2020 and the PRISMA-S (Preferred Reporting Items for Systematic Reviews and Meta-Analyses literature search extension) for reporting literature searches guidelines [80] (Multimedia Appendix 1). We also registered our review on PROSPERO (CRD20251056902). There were no deviations from the registered protocol.

### Information Sources and Search Strategy

To include as many studies of text-based ADE as possible at the beginning, we systematically searched 4 datasets: Scopus, IEEE Xplore, Web of Science, and PubMed. The search strategy followed the PRISMA-S reporting extension [81] and was developed based on common model architectures, text features, and outcome formats used in ADE research. The search strategy included combinations of the following terms: (“depression” OR “depressive” OR “clinical depression” OR “major depressive disorder” OR “MDD”) AND (“assessment” OR “measur*” OR “diagnos*” OR “predict*” OR “estimat*”) AND (“automated” OR “automatic” OR “AI” OR “machine learning” OR “deep learning” OR “large language model” OR “natural language processing” OR “NLP”) AND (“text” OR “linguistic analys*” OR “sentiment analys*” OR “semantic analys*” OR “lexical feature*” OR “transcribed speech” OR “written” OR “textual*”). The initial search was conducted on February 24, 2025, covering studies published from January 2014 onward, and was updated on December 3, 2025. Full database-specific search strings are provided in Multimedia Appendix 2. Additional eligible studies were identified by manually searching the reference lists of the included studies and previous reviews.

### Eligibility Criteria

We included studies that fulfill the following four criteria: (1) used texts to investigate depression, (2) used ML to establish an ADE model, (3) reported the data collection process and the depression label sources, and (4) reported the metrics of the model for effect size calculation. Our exclusion criteria are the studies that (1) did not use the standard depression label (details in paragraph 5 of the introduction); (2) were not written in English; (3) published before 2024 and having fewer citations than years of publish were excluded (eg, articles published in 2021 with 3 citations, or articles published in 2019 with 5 citations would be included) [31], to ensure that full-text screening focused on studies that had been acknowledged and referenced within the field; and (4) were developed exclusively on public datasets, as these models often iterate rapidly, which could introduce potential bias.

### Data Collection Process

Two independent researchers (SZ and JZ) excluded duplicates from the relevant studies retrieved, then screened and selected the relevant studies by inclusion and exclusion criteria. Disagreements were solved through discussion with a third author (CZ) when necessary. The relevant studies were evaluated through title and abstract screening. Thereafter, the remaining studies were screened through full texts to identify those for further analysis. Studies were managed using EndNote and Microsoft Excel. No automation tools were used.

### Data Items

Two researchers (SZ and JZ) independently coded the included studies in Microsoft Excel. The following information was extracted from each article: (1) study characteristics (first author and publication year), (2) annotation source (clinician diagnosis and self-report scales [PHQ-9]), (3) population characteristics (sample size and positive rate), (4) modeling strategy (text sources [eg, interviews, social media, writing tasks, and diaries], text representation [eg, RoBERTa, transformer, TF-IDF, LIWC, and Empath], model architecture [deep learning vs shallow ML] and validation strategies [eg, cross-validation, hold-out, and external validation]), and (5) predictor values [sensitivity, specificity, *F*_1_-score or data used to impute these values]. Furthermore, for studies that used different algorithms based on the same text dataset, only the model with the highest *F*_1_-score was included in the meta-analysis. If a study reported results based on different text sources (eg, clinical transcripts vs written documents), the best-performing model from each dataset was included separately. For multimodal studies, only the performance metrics from the text modality were included.

In addition, we assessed the reporting quality of each included study using the TRIPOD (Transparent Reporting of a multivariable prediction model for Individual Prognosis Or Diagnosis) tool [82]. TRIPOD was adapted to be more specific to NLP studies: The modifications were based on previously published research of NLP studies [83,84]. Two independent researchers (SZ and JZ) independently assessed each study based on TRIPOD items; disagreements were resolved through a third author (CZ).

### Effect Measures

For each study, the sensitivity, specificity, and sample sizes were used to calculate the effect size (ES). Specifically, the odds ratio (OR) was first computed by sensitivity and specificity, followed by a log transformation to obtain log (OR) [85]. The SE of log (OR) was then calculated based on the sensitivity, specificity, and sample size. Log (OR) and SE (log OR) were converted into ES by using Comprehensive Meta-Analysis (version 4; Biostat Inc). To direct comparison of model performance across studies, Pearson correlation coefficient (*r*), transformed via Fisher *z* for analysis and subsequently back-transformed for interpretation, was selected as the ES indicator [86]. Furthermore, the Pearson correlation coefficient contributes to a general understanding of the relationship between text-based models’ predictions and true labels. Values of *r* around 0.1, 0.3, and 0.5 are generally interpreted as indicating small, moderate, and large effect sizes, respectively. These thresholds have been widely applied in psychological and behavioral research, including prior reviews of depression prediction models [78,87].

### Data Synthesis and Analysis

Meta-analysis was initially performed using Comprehensive Meta-Analysis (version 4; Biostat Inc). Between-study heterogeneity was assessed using the chi-square *Q* statistic, which tests the null hypothesis of homogeneity [88]. The extent of heterogeneity was further described using the inconsistency index (*I*^2^), along with the between-study variance (τ²) and its SD (τ) [89]. In addition, 95% prediction intervals were reported to reflect the expected range of effects in future studies [90]. We adopted the random-effects model to assess the heterogeneity among studies and estimate the pooled effect size from each study. Given the limited number of included studies, the Hartung-Knapp-Sidik-Jonkman (HKSJ) method was applied to adjust the SEs [91]. Because CMA 4.0 does not implement the HKSJ procedure, we reestimated the pooled effects using the Meta package in R (version 4.3.2), and the 95% CI values reported in the main text were derived from the HKSJ-adjusted models.

To explore potential sources of heterogeneity, we performed a moderator analysis with a random effects model [92]. According to the extracted information from each study, four predefined groups were included: (1) text representation (embedding-based vs traditional features), (2) annotation source (clinician diagnosis vs self-reported scale), (3) model architecture (deep vs shallow), and (4) text source (documentation vs transcribed speech). For each subgroup, pooled ES (*r*) were estimated under a random-effects model, and between-group differences were assessed using the *Q*-test for heterogeneity. In addition, univariate meta-regression analyses were conducted to examine the potential moderating effects of 3 continuous covariates: reporting quality (TRIPOD score), sample size (log-transformed), and positive rate. Each covariate was analyzed separately with a random-effects model, and an analog *R^2^* indicated its explanatory power for study variance. Finally, we performed a sensitivity analysis using the leave-one-out method to assess the influence of each study on the overall results. We also conducted a cumulative meta-analysis to explore whether more recent studies have contributed to increased consistency.

### Small-Study Effects

We assessed potential small-study effects, including the possibility of publication bias, by visually inspecting a funnel plot of the SE Fisher *z* [93]. Statistical evaluation of funnel plot asymmetry was conducted using the Begg–Mazumdar rank correlation test and the Egger regression test, with a *P* value <.05 indicating significant small-study effects [94]. In addition, we applied the Duval and Tweedie trim-and-fill procedure to explore the potential impact of missing studies on the pooled effect size [95].

### Quality and Certainty Assessment

The methodological risk of bias of each included study was independently assessed by 2 authors (SZ and CZ) across five domains relevant to text-based ML research: (1) participant selection, (2) outcome labeling, (3) text acquisition and preprocessing, (4) model development and validation, and (5) reporting completeness. Each domain was rated as “low risk,” “unclear risk,” or “high risk” and an overall judgment was derived accordingly. The certainty of evidence for the primary pooled effect was evaluated using the GRADE (Grading of Recommendations Assessment, Development and Evaluation) framework [96], considering risk of bias, inconsistency, indirectness, imprecision, and publication bias.

## Results

### Study Selection Process

**Figure 1:**
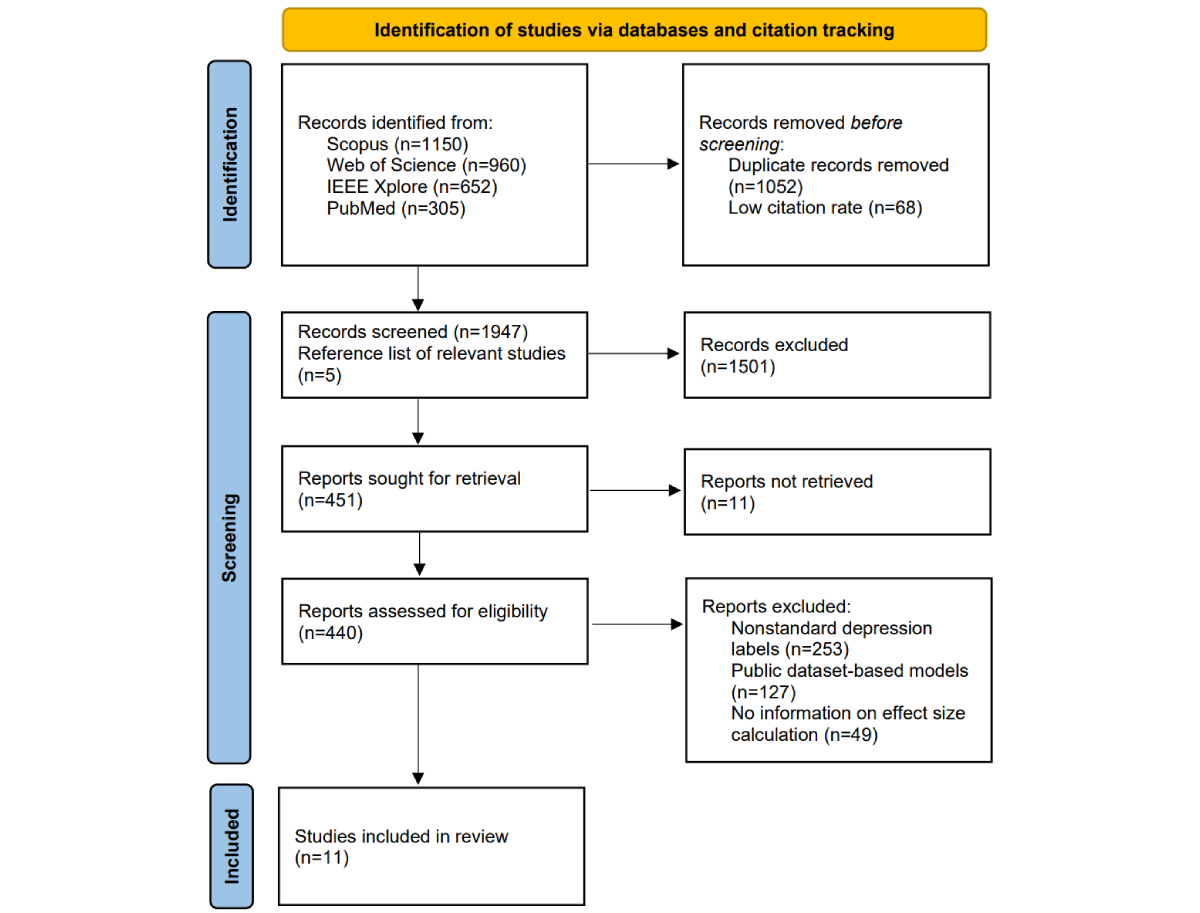
PRISMA (Preferred Reporting Items for Systematic Reviews and Meta-Analyses) 2020 flow diagram of studies included in this review.

The flow diagram of studies screening and selection is presented in Figure 1. The initial database search was conducted on February 24, 2025, and was updated on December 3, 2025.

A total of 3067 records were identified through database searching, including Web of Science (n=960), PubMed (n=305), Scopus (n=1150), and IEEE Xplore (n=652). After removing 1052 duplicates and 68 low-citation records, 1947 records remained for title and abstract screening, together with 5 additional records identified through manual citation tracking. Among them, 1501 records were excluded because they were not relevant to text-based depression estimation, including that the model inputs were not text data and no quantitative results were reported. The remaining 451 records were included in the full-text screening, and 11 studies that were inaccessible or retracted were first excluded. Next, we excluded studies that used nonstandard depression labels (n=253), studies based on public datasets (n=127), and studies lacking sufficient information to compute effect sizes (n=49). Finally, 11 studies [17,24,26,67,97-103] were included in the meta-analysis.

### Characteristics of Included Studies

A total of 11 studies were included in the final meta-analysis [17,24,26,67,97-103], contributing 15 independent text-based depression estimation models. A summary of included studies and model characteristics is presented in Table 1, and the detailed data extraction table is provided in Multimedia Appendix 3. The sample size ranged from 77 to 749 participants, with a median of 110. Positive rates (proportion of participants labeled as depressed) varied substantially across studies, ranging from 9.2% to 77.9%, reflecting differences in population selection and depression labeling strategies.

**Table 1:** Summary of study and model characteristics included in the meta-analysis (n=15).

Author (year)	Sample size^a^	Positive rateᵇ	TRIPODᶜ	Text representationᵈ	Annotation sourceᵉ	Model architectureᶠ	Text source^ᵍ^	Sensitivity	Specificity
Geraci et al (2017) [97]	366	0.243	17	Traditional features	Clinician diagnosis	Deep	Documentation	0.94	0.68
Ricard et al (2018) [26]	749	0.0921	13	Traditional features	Self-report scale	Shallow	Documentation	0.57	0.77
Tlachac et al (2020) [98]	162	0.3395	12	Traditional features	Self-report scale	Shallow	Documentation	0.93	0.63
Zhao et al (2021) [67]	110	0.4	11	Traditional features	Self-report scale	Shallow	Documentation	0.33	0.86
Zhao et al (2021) [67]	114	0.614	11	Traditional features	Self-report scale	Shallow	Documentation	0.81	0.53
Zhao et al (2021) [67]	341	0.463	11	Traditional features	Self-report scale	Shallow	Documentation	0.51	0.86
Shin et al (2022) [99]	166	0.5	16	Traditional features	Clinician diagnosis	Shallow	Transcribed speech	0.7	0.97
Cariola et al (2022) [24]	140	0.514	9	Traditional features	Clinician diagnosis	Shallow	Transcribed speech	0.68	0.66
Munthuli et al (2023) [100]	80	0.5	15	Embedding-based	Clinician diagnosis	Deep	Transcribed speech	0.83	0.85
Munthuli et al (2023) [100]	80	0.5	15	Embedding-based	Clinician diagnosis	Deep	Transcribed speech	0.88	0.93
Munthuli et al (2023) [100]	80	0.5	15	Embedding-based	Clinician diagnosis	Deep	Transcribed speech	0.9	0.83
Tlachac et al (2023) [101]	88	0.602	13	Traditional features	Self-report scale	Shallow	Documentation	0.79	0.74
Jihoon et al (2024) [102]	77	0.779	17	Traditional features	Clinician diagnosis	Shallow	Transcribed speech	0.96	0.25
Shin et al (2024) [17]	91	0.171	14	Embedding-based	Self-report scale	Deep	Documentation	0.929	0.761
Xu et al (2025) [103]	100	0.55	19	Embedding-based	Clinical diagnosis	Deep	Transcribed speech	0.955	0.933

^a^Validation strategies include k-fold cross-validation, leave-one-out, and nested validation.

ᵃSample size refers to the total number of participants included in each model.

ᵇPositive rate refers to the proportion of participants labeled as depressed.

ᶜTRIPOD score was based on a modified 27-item checklist adapted for NLP-based studies.

ᵈText representations include traditional features (eg, Lexical, TF-IDF, LWIC, and Emotional Dictionary) and embedding-based features (eg, BERT, Word2Vec, and RoBERTa).

ᵉAnnotation source indicates whether the depression label was derived from clinician diagnosis or self-report scales (Patient Health Questionnaire-9, PHQ-9).

ᶠModel architecture refers to shallow learning (eg, SVM, Logistic Regression, and Random Forests) versus deep learning (eg, GRU, BERT, Transformers).

ᵍText sources were classified as documentation (eg, Writing, Social media, and Messages) or transcribed speech (eg, Clinical Interviews and Psychological Conversations).

Regarding some characteristics of the modeling, 8 (53.3%) models used clinician diagnosis as depression labels, and the remaining 7 (46.7%) models relied on self-report scales such as the PHQ-9. In total, 10 (66.7%) models used traditional features to text representation, such as TF-IDF, LIWC, or emotional dictionaries, while 5 (33.3%) models used embedding-based techniques, including BERT and Word2Vec. 9 (60%) models used shallow learning models (eg, SVM, random forest, and logistic regression), and the remaining 6 (40%) models adopted deep learning architectures (eg, GRU and BERT-based classifiers). Text sources were classified as either documentation (n=8, 53.3%) or transcribed speech (n=7, 46.7%), the latter often derived from clinical interviews or therapeutic dialogues. Validation strategies also varied: 11 models implemented some form of k-fold cross-validation, while others applied nested or leave-group-out cross-validation. The reporting quality (mean 13.9, SD 2.8; range 9-19) of all included models was assessed using an adapted 21-item TRIPOD checklist for NLP-based predictive modeling. Detailed scoring criteria and individual scores are provided in Multimedia Appendix 4.

### Results of Meta-Analysis

**Figure 2:**
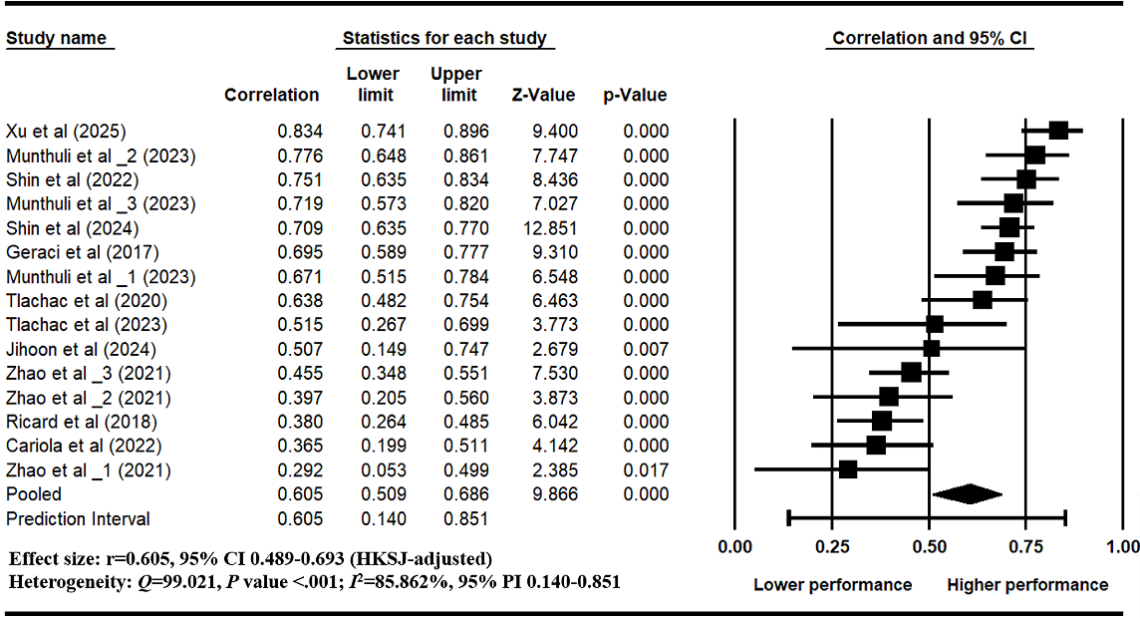
Forest plot presenting the pooled effect size (correlation r) of machine-learning models for text-based depression estimation trained with gold-standard labels [17,24,26,67,97-103]. For studies appearing more than once in the forest plot (eg, Zhao et al, 2021 [67]; Munthuli et al, 2023 [100]), indicate models trained and evaluated on independent text datasets derived from nonoverlapping participant samples.

A random-effects model was applied to synthesize the effect sizes of 15 text-based depression estimation models extracted from 11 eligible studies [17,24,26,67,97-103]. In studies reporting multiple models, each model was derived from a distinct text dataset with nonoverlapping participant samples and was therefore treated as a point estimate [104]. The pooled effect size (*r*) was 0.605 (95% CI 0.498-0.693) [87], indicating an overall high predictive ability in models using standard depression labels (Figure 2). Substantial between-model heterogeneity was observed (*Q*=99.02, *P*<.001; *I*²=85.9%). The estimated between-study variance was τ²=0.062 (τ=0.249). The 95% prediction interval ranged from 0.140 to 0.851, indicating considerable variability in the magnitude of effects expected across future studies. The forest plot revealed that each model achieved significant correlations (*P*<.05), the effect sizes varied across models (ranging from 0.292 to 0.776), further supporting the observed heterogeneity. The HKSJ-adjusted forest plot generated in R is provided in Multimedia Appendix 5. This variation highlights the influence of study-level characteristics such as model architecture, annotation source, and text representation on model performance, which were further analyzed in subsequent moderator and meta-regression analyses.

### Results of Subgroup Analysis for Moderators

**Figure 3:**
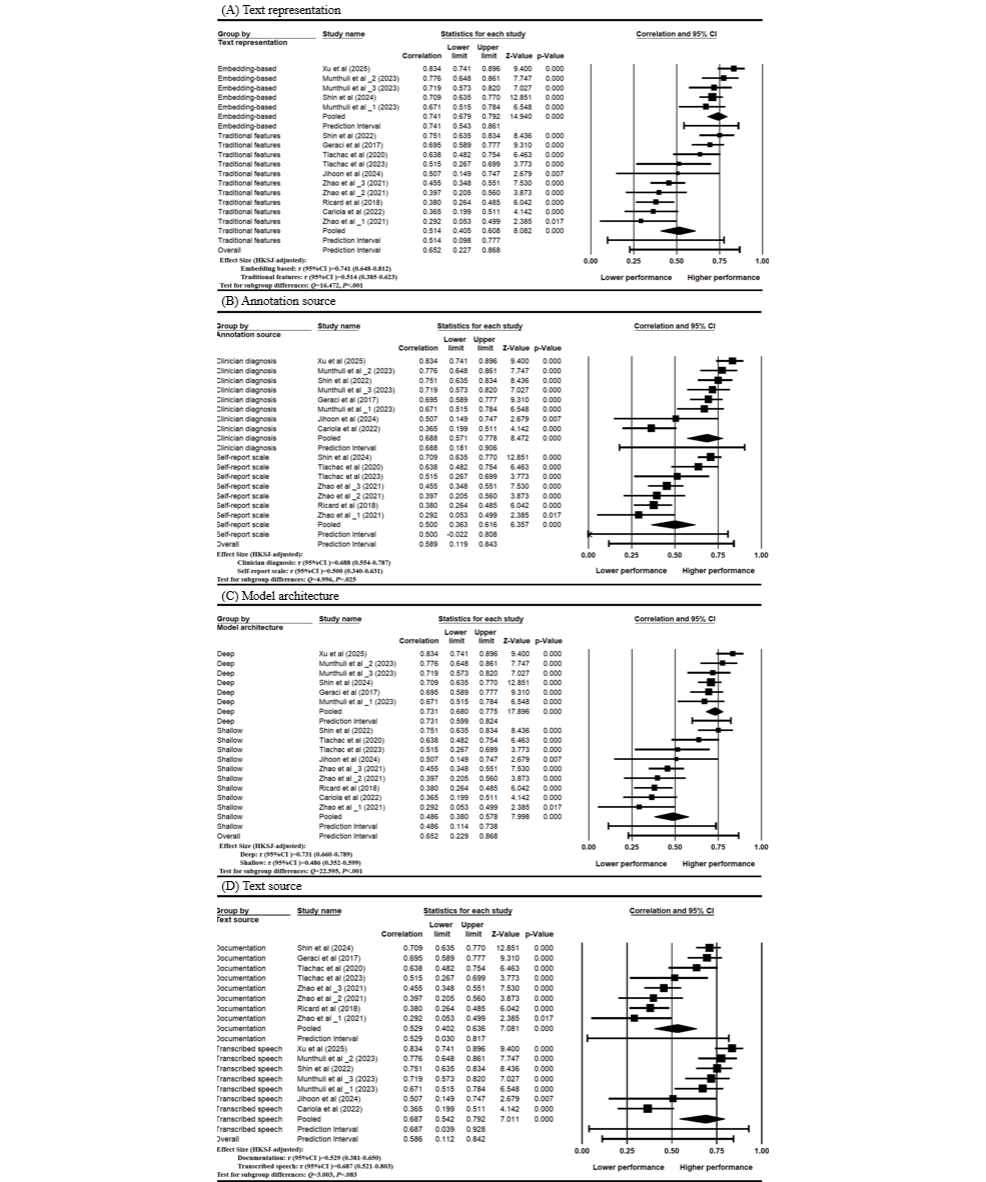
Forest plots of subgroup analyses by (A) text representation, (B) annotation source, (C) model architecture, and (D) text source generated using CMA4.0 [17, 24, 26, 67, 97, 98, 99, 100, 101, 102, 103]. High-resolution versions of all subgroup forest plots are provided in Multimedia Appendix 5.

Subgroup analyses were conducted to examine the moderating effects of text representation, annotation source, model architecture, and text source (Table 2). Significant moderation effects were observed for text representation (*Q*=16.47, *P*<.001) and model architecture (*Q*=22.60, *P*<.001). Models using embedding-based features yielded higher performance (*r*=0.741, 95% CI 0.648-0.812) compared with those using traditional features (*r*=0.514, 95% CI 0.385-0.623). Deep learning architectures also outperformed shallow models (*r*=0.731, 95% CI 0.660-0.789 vs *r*=0.486, 95% CI 0.352-0.599). Annotation source showed a statistically significant moderation effect (*Q*=5.00, *P*=.03), with models using clinician diagnoses achieving higher pooled performance (*r*=0.688, 95% CI 0.554-0.787) than those using self-report scales (*r*=0.500, 95% CI 0.340-0.631). Text source did not reach statistical significance (*Q*=3.00, *P*=.08), although models using transcribed speech tended to outperform those using documentation (r=0.687 vs 0.529). Forest plots of these subgroup analyses are presented in Figure 3, and HKSJ-adjusted subgroup plots generated in R are provided in Multimedia Appendix 5.

**Table 2:** Subgroup analyses examining the influence of text representation, annotation source, model architecture, and text source on model performance in text-based depression estimation.

Moderators		Models, n (%)	Point estimation (95% CI)	*Q*-value (*df*)	*P* value
**Text representation**		15 (100)	—^a^	16.472 (1)	<.001
	Embedding-based	5 (33.3)	0.741 (0.648-0.812)	—^a^	<.001
	Traditional features	10 (66.7)	0.514 (0.385-0.623)	—*	<.001
**Annotation source**		15 (100)	—^a^	4.996 (1)	.03
	Clinician diagnosis	8 (53.3)	0.688 (0.554-0.787)	—^a^	<.001
	Self-report scale	7 (46.7)	0.500 (0.340-0.631)	—^a^	<.001
**Model architecture**		15 (100)	—^a^	22.595 (1)	<.001
	Deep	6 (40)	0.731 (0.660-0.789)	—^a^	<.001
	Shallow	9 (60)	0.486 (0.352-0.599)	—^a^	<.001
**Text source**		15 (100)	—^a^	3.003 (1)	.08
	Documentation	8 (53.3)	0.529 (0.381-0.650)	—^a^	<.001
	Transcribed speech	7 (46.7)	0.687 (0.521-0.803)	—^a^	<.001

^a^Not applicable.

Univariable meta-regression analysis was used to test 3 continuous moderators: TRIPOD score, the positive rates, and the log-transformed sample size (log n). All meta-regressions were conducted using Fisher *z*-transformed correlation coefficients under a random-effects model. As shown in Table 3, only the TRIPOD score was significantly associated with model performance (β=0.085, 95% CI 0.050-0.119; *P*<.001), indicating a positive association between reporting quality and effect size. This model explained 71% of the between-study variance (*R²* analog=0.71). In contrast, neither the positive rate (β=–0.027, 95% CI –0.830 to 0.884; *P*=.95) nor the sample size (β=–0.012, 95% CI –0.214 to 0.190; *P*=.91) showed significant associations with effect size. Corresponding regression plots are included in Multimedia Appendix 6 (CMA-based) and Multimedia Appendix 5 (HKSJ-adjusted).

**Table 3:** Univariable meta-regression assessing reporting quality (TRIPOD), positive rate, and sample size as moderators of model performance.

Moderators	N	β (95% CI)	SE	*R*²	*P* value
TRIPOD^a^ NLP^b^	15	0.085 (0.050-0.119)	0.018	0.71	<.001
Positive Rate	15	–0.027 (–0.830 to 0.884)	0.437	<0.2	.95
Log n	15	–0.012 (–0.214-0.190)	0.103	<0.2	.91

^a^TRIPOD: Transparent Reporting of a multivariable prediction model for Individual Prognosis Or Diagnosis.

^b^NLP: natural language processing.

### Risk of Bias and Certainty Assessment

Risk-of-bias assessment showed that most included models demonstrated low methodological concerns. Of the 15 models, 7 were rated as low risk, 6 as unclear risk, and 2 as high risk, with the latter typically related to concerns in outcome measurement or reporting (Multimedia Appendix 7). The overall certainty of evidence for the primary pooled effect was rated as moderate under the GRADE framework. This rating was driven mainly by the substantial heterogeneity observed across models, while concerns regarding imprecision and publication bias were minimal. Detailed domain ratings and justification for GRADE decisions are provided in Supplementary Table S4 (Multimedia Appendix 7).

### Sensitivity Analysis and Cumulative Analysis

**Figure 4:**
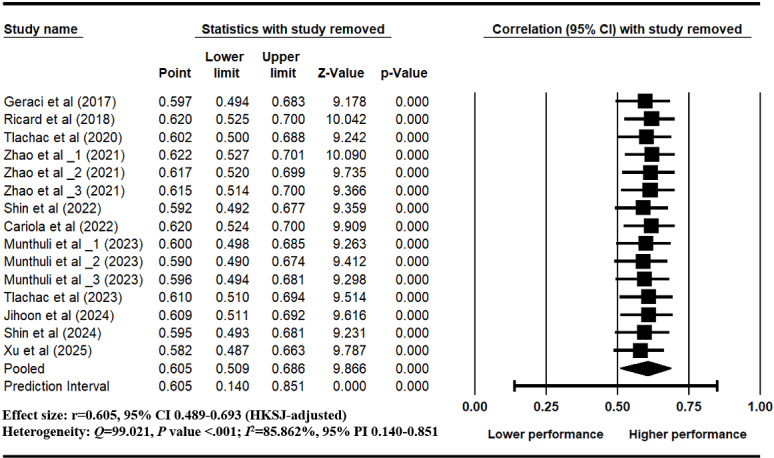
Leave-one-out sensitivity analysis [17,24,26,67,97-103].

**Figure 5:**
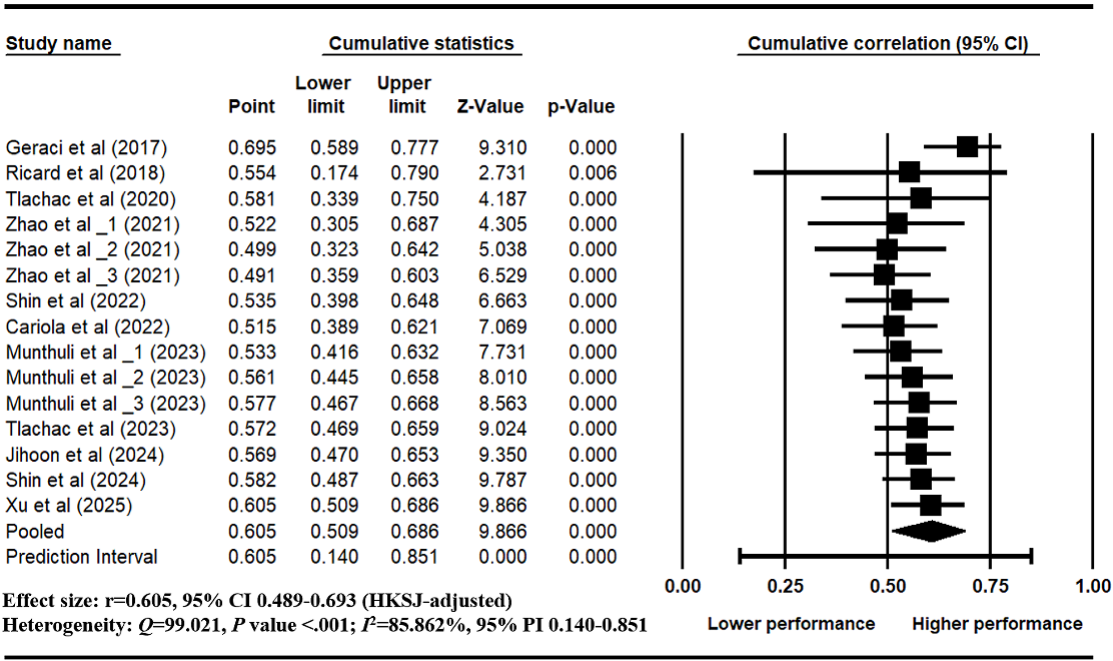
Cumulative meta-analysis [17,24,26,67,97-103].

Leave-one-out analysis indicated that the pooled effect size remained stable across all iterations (*r*=0.605, 95% CI 0.498-0.693), suggesting that no single study had a disproportionate impact on the overall result (Figure 4). Cumulative meta-analysis showed that the effect estimates gradually stabilized over time, indicating consistency across publication years (Figure 5).

### Small-Study Effects

**Figure 6:**
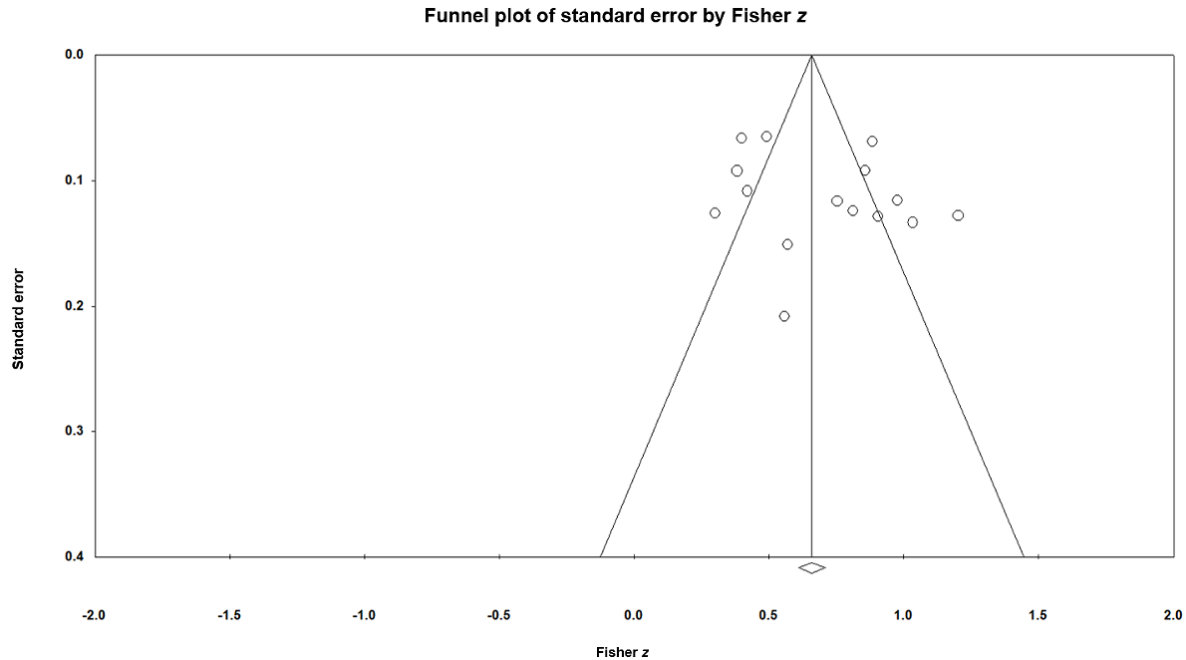
Funnel plot.

The funnel plot (Figure 6) showed a generally symmetrical distribution of effect sizes. Both the Begg–Mazumdar test (Kendall τ=0.17143, *P*=.37) and the Egger regression test (t_14_=1.13401, 2-tailed *P*=.28) were nonsignificant, indicating no evidence of small-study effects. The trim-and-fill procedure did not impute any additional studies, and the adjusted pooled effect was identical to the original estimate.

## Discussion

### Principal Findings

This study evaluated the performance of text-based depression estimation models that were trained and validated using standard depression labels. Our findings indicate that text-based ADE models demonstrate promising predictive performance. A previous review study mentioned the importance of a uniform standard label when evaluating the consistency of model performance across studies [78]. In this study, we focused on the studies using clinical diagnoses or PHQ-9 and excluded weak depression labels datasets (eg, keyword matching and self-declared), thereby enhancing the clinical reliability of the results [72,73]. These findings support the potential of text, especially that in transcribed speech and personal documentation, as informative signals for depression detection [23-25]. Nonetheless, the high between-study heterogeneity implies the differences in study-level characteristics, including modeling approaches, corpus sources, and reporting quality.

For the text representation and model architecture, the results of subgroup analyses showed that models using embedding-based text representations achieved higher performance than those using traditional features, and deep learning architectures outperformed shallow models. Consistent results have been reported on commonly used public benchmarks [105,106]. On the DAIC-WOZ dataset, the high-performing models often use embedding-based representation and deep model architecture [37,107]. This result aligns with the development of context-aware models like BERT [45] and GPT [46], which have superior semantic representation capabilities. A previous review study on emotion detection also reported similar conclusions [22]. However, the traditional linguistic features remain critical in the field of ADEs [16]. Linguistic features can be extracted from depressive texts by topic modeling techniques [108], which provide insights into underlying cognitive and emotional states and contribute to clinical understanding [109,110]. Overall, embedding-based and deep model architectures are superior in prediction performance, and the traditional features and shallow models remain valuable for enhancing interpretability and depression understanding.

For the annotation sources, the results of subgroup analyses indicated that models trained with clinician diagnoses also outperformed better than those relying on self-report scales. This supported the view expressed by Mao et al [31] that self-reported measures may not align with clinical diagnoses, potentially leading to inconsistency in model performance. Another study also highlighted the heterogeneity of depression estimation caused by subjectivity [111]. In addition, the results indicate that while models trained with clinically validated scales like PHQ-9 remain usable, their performance is generally lower than models trained with clinician diagnoses. For the text sources, models using transcribed speech were slightly higher than those using personal documentation, but this difference did not reach a statistically meaningful level. This may reflect the more information and affective cues embedded in spoken language [38], but some studies have pointed out that there are also noises such as stop words that are not directly related to emotion [112,113]. Overall, these results suggest that the annotation source demonstrated a determinative impact on model performance, with clinician diagnoses outperforming PHQ-9, whereas the text source showed only directional effects. We suggest constructing the ADE models based on the gold standard and natural environment-related text data. However, when clinician diagnoses are unavailable, validated self-report scales represent feasible alternatives.

It is worth noting that there is a significant positive correlation between the model report quality (TRIPOD score) and the model performance. This indicates that studies with higher report quality exhibit better performance in the ADE model. The TRIPOD checklist has been previously adopted in other automatic estimation domains [114,115]. This result further emphasized the necessity for comprehensive reporting of study-level characteristics, including modeling approaches and corpus sources, to enhance reliability and validity in ADE models [82]. In contrast, sample size and positive rates were not significantly associated with model performance. It is consistent with the previous review studies’ results [76,78], suggesting that in the ADE tasks, the data quality, annotation, and methodological transparency may be more crucial than the quantity of the samples. This finding underscores the importance of rigorous methodological reporting for ADE studies.

Taken together, the findings of this meta-analysis highlight several methodological and reporting considerations that are relevant for the development and evaluation of text-based ADEs. Specifically, models using embedding-based features and deep architectures are superior in predictive performance, suggesting that future studies could prioritize richer text representations combined with more sophisticated model architectures. Adopting standardized depression labels, particularly clinician diagnoses or widely validated scales, may further facilitate comparability across studies. Notably, we found that there is a significant positive correlation between the TRIPOD score and the model performance. This indicates that improving adherence to established reporting guidelines could enhance the transparency and reproducibility of ADE models. The overall certainty of evidence was rated as moderate using the GRADE approach, reflecting acceptable confidence in the pooled findings despite substantial between-study heterogeneity.

### Strengths and Limitations

The primary strength of this review lies in its strict focus on text-based ADE models trained and validated using standard depression labels. By excluding studies based on weak or inconsistent annotation sources, such as keyword matching or self-declared labels, we reduced label-related heterogeneity and improved the clinical interpretability of the synthesized findings. In addition, this meta-analysis systematically examined multiple methodological sources of heterogeneity, including text representation, model architecture, annotation source, and reporting quality, using a unified analytical framework combining subgroup analyses and meta-regression.

Nevertheless, this study also has some limitations. First, our inclusion criteria strictly require the use of standard depression labels. Although this enhanced the reliability of the labels, it also reduced the number of eligible studies (57.5% of the literature was excluded in the full-text assessment). Some of the latest studies were also not included, including large language models [116] and the prompt learning strategy [36]. Studies on conversational agents with NLP-based automatic depression detection were also not included [117]. Second, although the Egger test indicated no significant publication bias, the exclusion of non-English and low-visibility studies raises the possibility of study omission. Future work may cautiously broaden the inclusion scope to better capture emerging methodological trends, including hybrid approaches that integrate text with other modalities, with the aim of improving the practical applicability of ADE models.

### Implications

The results of this review indicate that multiple methodological factors, including text source, text representation, model architecture, annotation source, and reporting quality, influence the performance of text-based depression estimation models. These findings not only provide methodological guidance for future text-based ADE research but also offer important insights into the sources of variation observed in existing studies. To our knowledge, this study is among the first systematic reviews to explicitly restrict inclusion to models trained and validated using standard depression labels. Previous systematic reviews in this field have largely focused on aspects such as model architecture or the use of social media data, with little systematic attention given to the impact of depression label quality. The inclusion of weak depression labels in prior research may have affected the reliability of reported results and further limited their practical applicability. By treating standard depression labels as a core inclusion criterion, this review demonstrates that annotation source is one of the key determinants of model performance. This observation suggests that performance differences across studies may reflect variations in labeling standards and reporting quality, rather than differences in intrinsic model capability alone. More broadly, the present findings support a shift in text-based ADE research toward evaluation frameworks that emphasize label rigor, transparent reporting, and methodological consistency, thereby enabling more meaningful comparison across studies and ultimately facilitating the clinical translation of ADE models.

### Conclusions

In summary, this systematic review and meta-analysis surveyed the last decade of text-based ADE using standard depression labels. The overall pooled effect size (*r*=0.605) suggests that ADE models perform well and have the potential for practical application. However, substantial heterogeneity across studies was observed. Models using embedding-based features and deep architectures generally achieved superior performance, whereas the influence of annotation source and text source was comparatively limited. Models trained with clinical diagnoses and transcribed speech tended to outperform those using self-report scales and documentation, though the difference was not statistically significant. Moreover, reporting quality, as assessed by TRIPOD, was positively associated with performance, highlighting the need for transparent reporting. Future studies should consider richer text representations, standardized labels, integration with other modalities, and transparent reporting to enhance the reliability and practical applicability of ADE models. In our future work, we will also aim to capture emerging approaches and cautiously broaden the inclusion scope, thereby providing a more comprehensive view of the ADE field.

## Data Availability

All data and search strategies are available in the additional files accompanying this article. Further details in this study are available from the corresponding author upon reasonable request.

## Appendix

Multimedia Appendix 1 PRISMA 2020 checklist.

Multimedia Appendix 2 Search strategies.

Multimedia Appendix 3 Detailed data extraction table of included studies.

Multimedia Appendix 4 TRIPOD-AI_checklist.

Multimedia Appendix 5 Hartung–Knapp–Sidik–Jonkman corrected analyses using the R meta package.

Multimedia Appendix 6 CMA4.0-based subgroup and meta-regression analyses.

Multimedia Appendix 7 Risk of Bias and GRADE Assessment Tables.

## Abbreviations

ADE: automatic depression estimation
BDI-II: Beck Depression Inventory-II
BERT: Bidirectional Encoder Representations from Transformers
CES-D: Center for Epidemiological Studies Depression Scale
DAIC-WOZ: Distress Analysis Interview Corpus – Wizard-of-Oz
DASS-21: 21-item Depression, Anxiety, and Stress Scale
ES: effect size
GRADE: Grading of Recommendations Assessment, Development and Evaluation
HKSJ: Hartung-Knapp-Sidik-Jonkman
ML: machine learning
NLP: natural language processing
OR: odds ratio
PHQ-8: Patient Health Questionnaire-8
PHQ-9: Patient Health Questionnaire-9
PRISMA: Preferred Reporting Items for Systematic Reviews and Meta-Analyses
PRISMA-S: Preferred Reporting Items for Systematic Reviews and Meta-Analyses literature search extension
SDS: Self-Rating Depression Scale
TRIPOD: Transparent Reporting of a multivariable prediction model for Individual Prognosis Or Diagnosis
