# Supplementary material for: Text-Based Depression Estimation Using Machine Learning With Standard Labels: Systematic Review and Meta-Analysis
Source: J Med Internet Res. 2026 Feb 11;28:e82686. doi: 10.2196/82686 (PMC12936666; doi:10.2196/82686)
Supplement: Multimedia Appendix 2 [file jmir_v28i1e82686_app2.docx]

**Search Strategies**

**Platform: Clarivate
Database 1: Web of Science Core Collection**
**Search date:** December 2, 2025
**Search string:**

(TS = ((depression OR depressive OR "major depressive disorder" OR "clinical depression" OR "MDD") AND ("assessment" OR "measur*" OR "diagnos*" OR "predict*" OR "estimat*") AND ("automated" OR "automatic" OR "AI" OR "machine learning" OR "deep learning" OR "large language model" OR "natural language processing" OR "NLP") AND ("text" OR "linguistic analys*" OR "sentiment analys*" OR "semantic analys*" OR "lexical feature*" OR "textual*" OR "written" OR "transcribed speech"))

AND PY = (2014-2025)

AND LA = (English)) AND DT = (Article OR Proceedings Paper)

**Platform: Elsevier
Database 2: Scopus
Search date:** December 3, 2025
**Search string:**

TITLE-ABS-KEY ( ( "depression" OR "depressive" OR "clinical depression" OR "major depressive disorder" OR "MDD" ) AND ( "assessment" OR "measur*" OR "diagnos*" OR "predict*" OR "estimat*" ) AND ( "automated" OR "automatic" OR "ai" OR "machine learning" OR "deep learning" OR "large language model" OR "natural language processing" OR "nlp" ) AND ( "text" OR "linguistic analys*" OR "sentiment analys*" OR "semantic analys*" OR "lexical feature*" OR "textual*" OR "written" OR "transcribed speech") ) AND PUBYEAR > 2013 AND PUBYEAR < 2025 AND ( LIMIT-TO ( DOCTYPE,"ar" ) OR LIMIT-TO ( DOCTYPE,"cp" ) ) AND ( LIMIT-TO ( LANGUAGE,"English" ) )

**Platform: NLM
Database 3: PubMed
Search date:** December 3, 2025
**Search string:**

(("depression"[Title/Abstract] OR "depressive"[Title/Abstract] OR "clinical depression"[Title/Abstract] OR "major depressive disorder"[Title/Abstract] OR "MDD"[Title/Abstract]) AND ("assessment"[Title/Abstract] OR "measur*"[Title/Abstract] OR "diagnos*"[Title/Abstract] OR "predict*"[Title/Abstract] OR "estimat*"[Title/Abstract]) AND ("automated"[Title/Abstract] OR "automatic"[Title/Abstract] OR "ai"[Title/Abstract] OR "machine learning"[Title/Abstract] OR "deep learning"[Title/Abstract] OR "large language model"[Title/Abstract] OR "natural language processing"[Title/Abstract] OR "nlp"[Title/Abstract]) AND ("text"[Title/Abstract] OR "linguistic analys*"[Title/Abstract] OR "sentiment analys*"[Title/Abstract] OR "semantic analys*"[Title/Abstract] OR "lexical feature*"[Title/Abstract] OR "textual*"[Title/Abstract] OR "written"[Title/Abstract] OR "transcribed speech"[Title/Abstract])) AND ((fft[Filter]) AND (english[Filter]) AND (2014:2026[pdat]))

**Platform: IEEE Xplore
Database 4: IEEE Xplore Digital Library** **Search date:** December 3, 2025
**Search string:**

("All Metadata":": "depression" OR "depressive" OR "clinical depression" OR "major depressive disorder" OR "MDD") AND ("All Metadata":"assessment" OR "measur*" OR "diagnos*" OR "predict*" OR "estimat*") AND ("All Metadata": "automated" OR "automatic" OR "ai" OR "machine learning" OR "deep learning" OR "large language model" OR "natural language processing" OR "nlp") AND ( "All Metadata": "text" OR "linguistic analys*" OR "sentiment analys*" OR "semantic analys*" OR "lexical feature*" OR "textual*" OR "written" OR "transcribed speech")

**Filters applied:**

Publication Years 2014–2025; IEEE; English; Journals & Conferences.
