# Supplementary material for: Text-Based Depression Estimation Using Machine Learning With Standard Labels: Systematic Review and Meta-Analysis
Source: J Med Internet Res. 2026 Feb 11;28:e82686. doi: 10.2196/82686 (PMC12936666; doi:10.2196/82686)
Supplement: Multimedia Appendix 3 [file jmir_v28i1e82686_app3.docx]

Supplementary Table S2. Detailed data extraction table for all included studies.

| Study Characteristics | | |  | Population Characteristics | |  |  |  |  | Predictor Values | | |
| --- | --- | --- | --- | --- | --- | --- | --- | --- | --- | --- | --- | --- |
| Author (Year) | Country | Journal | Annotation Source | Sample  Size | Positive Cases | Text Source | Text Representation | Model Architecture | Validation Strategies | Sensitivity | Specificity | F1-score |
| Geraci et al (2017) | Canada | BMJ Mental Health | DSM-IV diagnosis annotated by two psychiatrists | 366 | 89 | Electronic Medical Records (EMR) – unstructured clinical notes from youth psychiatric patients | TF-IDF from Document Term Matrix | Feedforward Deep Neural Network | Fivefold Cross-validation + External validation | 0.94 | 0.68 | 0.72 |
| Ricard et al (2018) | United States | Journal of medical Internet research | PHQ-8（Patient Health Questionnaire-8） | 749 | 69 | Social media - Instagram post captions and comments | ANEW scores, LabMT scores, Emoji sentiment scores, plus metadata (e.g., number of likes, comments) | Elastic-net regularized linear regression | Hold-out validation（10% held-out independent test set + internal cross-validation） | 0.57 | 0.77 | 0.66 |
| Tlachac et al (2020) | United States | IEEE journal of biomedical and health informatics | PHQ-9 | 162 | 55 | Private Text Messages | Word category frequencies via Empath, POS frequencies, Sentiment, Volume features | Logistic Regression with PCA | 5-fold Cross-validation (repeated 100 times) | 0.93 | 0.63 | 0.81 |
| Zhao et al _1 (2021) | United States | IEEE Transactions on Affective Computing | PHQ-9 | 110 | 44 | Interview Transcript | LIWC-like lexical categories, POS tags, character/word count | XGBoost with Bayesian Optimization | Monte Carlo Cross-validation（25 iterations） | 0.33 | 0.86 | 0.44 |
| Zhao et al _2 (2021) | United States | IEEE Transactions on Affective Computing | PHQ-9 | 114 | 70 | SMS Text Messages | fiction5f lexical categories, text count | XGBoost with Bayesian Optimization | Monte Carlo Cross-validation（25 iterations） | 0.81 | 0.53 | 0.76 |
| Zhao et al _3 (2021) | United States | IEEE Transactions on Affective Computing | PHQ-9 | 341 | 158 | Typed Replies | Empath lexical categories, POS, character/word count | XGBoost with Bayesian Optimization | Monte Carlo Cross-validation（25 iterations） | 0.51 | 0.86 | 0.61 |
| Shin et al  (2022) | Republic of Korea | Frontiers in psychiatry | MINI Diagnostic Interview + PHQ-9 + HDRS | 166 | 83 | Mini-International Neuropsychiatric Interview,MINI | Word Frequency + POS Tagging, (Konlpy） | Naive Bayes Classifier | Cross-validation（5-fold） | 0.7 | 0.97 | NR |
| Cariola et al (2022) | Scotland | International Conference on Affective Computing and Intelligent Interaction | SCID (Structured Clinical Interview for DSM-IV) + PHQ-9 | 140 | 72 | Mother - Adolescent Problem-Solving Task Transcript | LIWC: Focus Present, Focus Future, Focus Past, First-person pronouns, Second-person pronouns） | Support Vector Machine, SVM with SHAP-selected features | Leave-One-Out Cross-validation with nested grid search | 0.68 | 0.66 | NR |
| Munthuli et al _1 (2023) | Thailand | PLoS one | DSM-V + PHQ-9+ HAM-D | 80 | 40 | Spoken transcription text of the Thai depression assessment task | XLM-RoBERTa multilingual pre-trained embeddings | XLM-RoBERTaBASE | Stratified Nested Cross-validation（5×8 folds） | 0.83 | 0.85 | 0.84 |
| Munthuli et al _2 (2023) | Thailand | PLoS one | DSM-V + PHQ-9+ HAM-D | 80 | 40 | Spoken transcription text of the Thai depression assessment task | XLM-RoBERTa multilingual pre-trained embeddings | XLM-RoBERTaBASE + Averaging Ensemble | Stratified Nested Cross-validation（5×8 folds） | 0.88 | 0.93 | 0.90 |
| Munthuli et al _3 (2023) | Thailand | PLoS one | DSM-V + PHQ-9+ HAM-D | 80 | 40 | Spoken transcription text of the Thai depression assessment task | XLM-RoBERTa multilingual pre-trained embeddings | XLM-RoBERTaBASE + Averaging Ensemble | Stratified Nested Cross-validation（5×8 folds） | 0.9 | 0.83 | 0.86 |
| Tlachac et al (2023) | United States | IEEE Journal of Biomedical and Health Informatics | PHQ-9 | 88 | 53 | SMS Text Messages | Lexical category frequency features from automatically constructed lexicons—Fiction5f | Linear SVC, Polynomial SVC, Logistic Regression, Naive Bayes, KNN | Leave-group-out Cross-validation (100 iterations with replacement, stratified by label) | 0.79 | 0.74 | 0.79 |
| Jihoon et al (2024) | United States | Frontiers in psychiatry | DSM-5（Clinical diagnosis established through DSM-5 by two or more psychiatrists in person） | 77 | 60 | Psychiatric interviews transcribed to text, first 15-20 minutes of one-on-one psychiatrist-patient sessions | Probability vectors of eight emotions per sentence using Acryl Inc.'s Emotional Analysis Module | XGBoost classifier | Cross-validation（4-fold cross-validation on training set with 80/20 train-test split） | 0.96 | 0.25 | 0.88 |
| Shin et al  (2024) | Republic of Korea | Journal of Medical Internet Research | PHQ-9 | 91 | 16 | The semi-structured diary texts generated by users and obtained through the "Mind Station" emotional diary app | pre-trained large language models, | GPT-3.5 (Not fine-tuned, directly called using the OpenAI API) | Stratified 5-fold Cross-validation | 0.929 | 0.761 | 0.607 |
| Xu et al  (2025) | China | Journal of Affective Disorders | Clinical diagnosis through psychiatrists | 100 | 55 | Multi-round Chatbot Interview | XLNet encoder | XLNet encoder + FC classifier | Fivefold Cross-validation + External validation (independent test set) | 0.955 | 0.933 | 0.948 |
