# Supplementary material for: Text-Based Depression Estimation Using Machine Learning With Standard Labels: Systematic Review and Meta-Analysis
Source: J Med Internet Res. 2026 Feb 11;28:e82686. doi: 10.2196/82686 (PMC12936666; doi:10.2196/82686)
Supplement: Multimedia Appendix 4 [file jmir_v28i1e82686_app4.docx]

**Scoring of Included Studies Based on an Adapted TRIPOD-AI Checklist**

[Geraci et al. (2017). Applying Deep Neural Networks to Unstructured Text Notes in Electronic Medical Records for Phenotyping Youth Depression. 1](#_Toc216267265)

[Ricard et al. (2018). Exploring the Utility of Community-Generated Social Media Content for Detecting Depression: An Analytical Study on Instagram. 2](#_Toc216267266)

[Tlachac et al. (2020). Screening for Depression with Retrospectively Harvested Private Versus Public Text. 3](#_Toc216267268)

[Zhao et al _1. (2021). Bayesian Optimization with Tree Ensembles to Improve Depression Screening on Textual Datasets. 4](#_Toc216267269)

[Zhao et al _2. (2021). Bayesian Optimization with Tree Ensembles to Improve Depression Screening on Textual Datasets. 5](#_Toc216267271)

[Zhao et al _3. (2021). Bayesian Optimization with Tree Ensembles to Improve Depression Screening on Textual Datasets. 6](#_Toc216267272)

[Shin et al. (2022). Detection of Depression and Suicide Risk Based on Text From Clinical Interviews Using Machine Learning: Possibility of a New Objective Diagnostic Marker. 7](#_Toc216267273)

[Cariola et al. (2022). Language Use in Mother-Adolescent Dyadic Interaction: Preliminary Results. 8](#_Toc216267274)

[Munthuli et al _1. (2023). Classification and Analysis of Text Transcription from Thai Depression Assessment Tasks among Patients with Depression. 9](#_Toc216267275)

[Munthuli et al _2. (2023). Classification and Analysis of Text Transcription from Thai Depression Assessment Tasks among Patients with Depression. 10](#_Toc216267276)

[Munthuli et al _3. (2023). Classification and Analysis of Text Transcription from Thai Depression Assessment Tasks among Patients with Depression. 11](#_Toc216267277)

[Tlachac et al. (2023). Automated Construction of Lexicons to Improve Depression Screening With Text Messages. 12](#_Toc216267278)

[Jihoon et al. (2024). Development of Depression Detection Algorithm Using Text Scripts of Routine Psychiatric Interview. 13](#_Toc216267279)

[Shin et al. (2024). Using Large Language Models to Detect Depression From User-Generated Diary Text Data as a Novel Approach in Digital Mental Health Screening: Instrument Validation Study. 14](#_Toc216267280)

[Xu et al. (2025). Deep learning-based detection of depression by fusing auditory, visual and textual clues. 15](#_Toc216267281)

Geraci et al. (2017). Applying Deep Neural Networks to Unstructured Text Notes in Electronic Medical Records for Phenotyping Youth Depression.

| **Item** | **Section / Topic** | **Page NO.** |
| --- | --- | --- |
| **Title and Abstract** |  |  |
| Title | 1. Identify the study as developing and/or validating a natural language processing system, the target population, and the primary outcome. | 83 |
| Abstract | 1. Provide a summary of objectives, study design, setting, participants, sample size, outcome, statistical analysis, results, and conclusions | 83 |
| **Introduction** |  |  |
| Background and objectives | 1. Explain the medical context (including whether diagnostic or prognostic) and rationale for developing and validating the natural language processing system, including references to existing systems. | 83,84 |
|  | 1. Specify the objectives, including whether the study describes the development or validation of the model or both. | 83,84 |
| **Methods** |  |  |
| Source of data | 1. Describe the study design or source of data (e.g. internal or external datasets) and cohort characteristics for the training, development, and validation datasets, if applicable | 84,85,86 |
|  | 1. Specify key elements of the study setting (e.g. primary care, secondary care, general population) including number and location of centers. | 84 |
|  | 1. Describe what types of texts were included and why. | 84 |
| Outcome | 1. Clearly define the outcome determined by the system. | 85,86 |
|  | 1. Clearly define the reference standard (e.g. manual review of notes, ICD codes). | - |
| Statistical analysis methods | 1. Specify the type of system, including whether it was self-developed or an existing system, all system-building procedures, and method for internal validation. | 84,85,86 |
|  | 1. Specify all measures used to assess system performance and, if relevant, to compare multiple systems. Require characteristics include the training population, whether cross-validation was employed, the testing population, and whether a completely orthogonal cohort (for example, a different hospital) was used as an independent test. | 85,86,87 |
| Development vs. validation | 1. For validation, identify any differences from the development data in setting, eligibility criteria, or outcome. | - |
| **Result** |  |  |
| System development | 1. Specify the number of included texts in each analysis. | 84,85 |
| System performance | 1. Report performance measures. If the approach is predictive, accuracy, area under the curve, sensitivity, and specificity should be provided along with confidence intervals if relevant. | 85,86,87 |
| System update | 1. Perform an error analysis. Describe any system updating arising from the validation, if done. | - |
| **Discussion** |  |  |
| Limitations | 1. Discuss any limitations of the study (such as biased sample or lack of external validation). | 87 |
| Interpretation | 1. For validation, discuss the results with reference to performance in the development data, or any other validation data. | 85,86 |
|  | 1. Give an overall interpretation of results, considering objectives, limitations, results from similar studies, and other relevant evidence. | 86,87 |
| Implications | 1. Discuss the potential clinical use of the system and implications for future research. | 85,86,87 |
| **Other information** |  |  |
| Supplementary information | 1. Provide information about the availability of supplementary resources, such as study protocol, datasets, and source code. | - |
| Funding | 1. Give the source of funding and the role of the funders for the present study. | 87 |
| **Total Score: 17** |  |  |

Ricard et al. (2018). Exploring the Utility of Community-Generated Social Media Content for Detecting Depression: An Analytical Study on Instagram.

| **Item** | **Section / Topic** | **Page NO.** |
| --- | --- | --- |
| **Title and Abstract** |  |  |
| Title | 1. Identify the study as developing and/or validating a natural language processing system, the target population, and the primary outcome. | - |
| Abstract | 1. Provide a summary of objectives, study design, setting, participants, sample size, outcome, statistical analysis, results, and conclusions | 1 |
| **Introduction** |  |  |
| Background and objectives | 1. Explain the medical context (including whether diagnostic or prognostic) and rationale for developing and validating the natural language processing system, including references to existing systems. | 1,2,3 |
|  | 1. Specify the objectives, including whether the study describes the development or validation of the model or both. | 2,3 |
| **Methods** |  |  |
| Source of data | 1. Describe the study design or source of data (e.g. internal or external datasets) and cohort characteristics for the training, development, and validation datasets, if applicable | 2,3,4 |
|  | 1. Specify key elements of the study setting (e.g. primary care, secondary care, general population) including number and location of centers. | - |
|  | 1. Describe what types of texts were included and why. | 2,3 |
| Outcome | 1. Clearly define the outcome determined by the system. | 5 |
|  | 1. Clearly define the reference standard (e.g. manual review of notes, ICD codes). | 2 |
| Statistical analysis methods | 1. Specify the type of system, including whether it was self-developed or an existing system, all system-building procedures, and method for internal validation. | - |
|  | 1. Specify all measures used to assess system performance and, if relevant, to compare multiple systems. Require characteristics include the training population, whether cross-validation was employed, the testing population, and whether a completely orthogonal cohort (for example, a different hospital) was used as an independent test. | 3,4 |
| Development vs. validation | 1. For validation, identify any differences from the development data in setting, eligibility criteria, or outcome. | - |
| **Result** |  |  |
| System development | 1. Specify the number of included texts in each analysis. | - |
| System performance | 1. Report performance measures. If the approach is predictive, accuracy, area under the curve, sensitivity, and specificity should be provided along with confidence intervals if relevant. | 5,8 |
| System update | 1. Perform an error analysis. Describe any system updating arising from the validation, if done. | - |
| **Discussion** |  |  |
| Limitations | 1. Discuss any limitations of the study (such as biased sample or lack of external validation). | 9 |
| Interpretation | 1. For validation, discuss the results with reference to performance in the development data, or any other validation data. | - |
|  | 1. Give an overall interpretation of results, considering objectives, limitations, results from similar studies, and other relevant evidence. | 7 - 9 |
| Implications | 1. Discuss the potential clinical use of the system and implications for future research. | 8,9 |
| **Other information** |  |  |
| Supplementary information | 1. Provide information about the availability of supplementary resources, such as study protocol, datasets, and source code. | - |
| Funding | 1. Give the source of funding and the role of the funders for the present study. | 12 |
| **Total Score: 13** |  |  |

Tlachac et al. (2020). Screening for Depression with Retrospectively Harvested Private Versus Public Text.

| **Item** | **Section / Topic** | **Page NO.** |
| --- | --- | --- |
| **Title and Abstract** |  |  |
| Title | 1. Identify the study as developing and/or validating a natural language processing system, the target population, and the primary outcome. | - |
| Abstract | 1. Provide a summary of objectives, study design, setting, participants, sample size, outcome, statistical analysis, results, and conclusions | - |
| **Introduction** |  |  |
| Background and objectives | 1. Explain the medical context (including whether diagnostic or prognostic) and rationale for developing and validating the natural language processing system, including references to existing systems. | 3326,3327 |
|  | 1. Specify the objectives, including whether the study describes the development or validation of the model or both. | 3327 |
| **Methods** |  |  |
| Source of data | 1. Describe the study design or source of data (e.g. internal or external datasets) and cohort characteristics for the training, development, and validation datasets, if applicable | 3327,3328 |
|  | 1. Specify key elements of the study setting (e.g. primary care, secondary care, general population) including number and location of centers. | - |
|  | 1. Describe what types of texts were included and why. | 3327,3328 |
| Outcome | 1. Clearly define the outcome determined by the system. | 3329-3331 |
|  | 1. Clearly define the reference standard (e.g. manual review of notes, ICD codes). | - |
| Statistical analysis methods | 1. Specify the type of system, including whether it was self-developed or an existing system, all system-building procedures, and method for internal validation. | - |
|  | 1. Specify all measures used to assess system performance and, if relevant, to compare multiple systems. Require characteristics include the training population, whether cross-validation was employed, the testing population, and whether a completely orthogonal cohort (for example, a different hospital) was used as an independent test. | 3328,3329 |
| Development vs. validation | 1. For validation, identify any differences from the development data in setting, eligibility criteria, or outcome. | - |
| **Result** |  |  |
| System development | 1. Specify the number of included texts in each analysis. | - |
| System performance | 1. Report performance measures. If the approach is predictive, accuracy, area under the curve, sensitivity, and specificity should be provided along with confidence intervals if relevant. | 3328-3331 |
| System update | 1. Perform an error analysis. Describe any system updating arising from the validation, if done. | - |
| **Discussion** |  |  |
| Limitations | 1. Discuss any limitations of the study (such as biased sample or lack of external validation). | 3332 |
| Interpretation | 1. For validation, discuss the results with reference to performance in the development data, or any other validation data. | - |
|  | 1. Give an overall interpretation of results, considering objectives, limitations, results from similar studies, and other relevant evidence. | 3331,3332 |
| Implications | 1. Discuss the potential clinical use of the system and implications for future research. | 3331,3332 |
| **Other information** |  |  |
| Supplementary information | 1. Provide information about the availability of supplementary resources, such as study protocol, datasets, and source code. | 3329 |
| Funding | 1. Give the source of funding and the role of the funders for the present study. | 3326 |
| **Total Score: 12** |  |  |

Zhao et al _1. (2021). Bayesian Optimization with Tree Ensembles to Improve Depression Screening on Textual Datasets.

| **Item** | **Section / Topic** | **Page NO.** |
| --- | --- | --- |
| **Title and Abstract** |  |  |
| Title | 1. Identify the study as developing and/or validating a natural language processing system, the target population, and the primary outcome. | - |
| Abstract | 1. Provide a summary of objectives, study design, setting, participants, sample size, outcome, statistical analysis, results, and conclusions | - |
| **Introduction** |  |  |
| Background and objectives | 1. Explain the medical context (including whether diagnostic or prognostic) and rationale for developing and validating the natural language processing system, including references to existing systems. | 1,2,3 |
|  | 1. Specify the objectives, including whether the study describes the development or validation of the model or both. | 2,3,5-10 |
| **Methods** |  |  |
| Source of data | 1. Describe the study design or source of data (e.g. internal or external datasets) and cohort characteristics for the training, development, and validation datasets, if applicable | 3,4 |
|  | 1. Specify key elements of the study setting (e.g. primary care, secondary care, general population) including number and location of centers. | - |
|  | 1. Describe what types of texts were included and why. | 3,4 |
| Outcome | 1. Clearly define the outcome determined by the system. | 5-10 |
|  | 1. Clearly define the reference standard (e.g. manual review of notes, ICD codes). | - |
| Statistical analysis methods | 1. Specify the type of system, including whether it was self-developed or an existing system, all system-building procedures, and method for internal validation. | - |
|  | 1. Specify all measures used to assess system performance and, if relevant, to compare multiple systems. Require characteristics include the training population, whether cross-validation was employed, the testing population, and whether a completely orthogonal cohort (for example, a different hospital) was used as an independent test. | 6,7 |
| Development vs. validation | 1. For validation, identify any differences from the development data in setting, eligibility criteria, or outcome. | - |
| **Result** |  |  |
| System development | 1. Specify the number of included texts in each analysis. | 3,4 |
| System performance | 1. Report performance measures. If the approach is predictive, accuracy, area under the curve, sensitivity, and specificity should be provided along with confidence intervals if relevant. | 7,8 |
| System update | 1. Perform an error analysis. Describe any system updating arising from the validation, if done. | - |
| **Discussion** |  |  |
| Limitations | 1. Discuss any limitations of the study (such as biased sample or lack of external validation). | 10 |
| Interpretation | 1. For validation, discuss the results with reference to performance in the development data, or any other validation data. | - |
|  | 1. Give an overall interpretation of results, considering objectives, limitations, results from similar studies, and other relevant evidence. | 10,11 |
| Implications | 1. Discuss the potential clinical use of the system and implications for future research. | 9,10 |
| **Other information** |  |  |
| Supplementary information | 1. Provide information about the availability of supplementary resources, such as study protocol, datasets, and source code. | - |
| Funding | 1. Give the source of funding and the role of the funders for the present study. | - |
| **Total Score: 11** |  |  |

Zhao et al _2. (2021). Bayesian Optimization with Tree Ensembles to Improve Depression Screening on Textual Datasets.

| **Item** | **Section / Topic** | **Page NO.** |
| --- | --- | --- |
| **Title and Abstract** |  |  |
| Title | 1. Identify the study as developing and/or validating a natural language processing system, the target population, and the primary outcome. | - |
| Abstract | 1. Provide a summary of objectives, study design, setting, participants, sample size, outcome, statistical analysis, results, and conclusions | - |
| **Introduction** |  |  |
| Background and objectives | 1. Explain the medical context (including whether diagnostic or prognostic) and rationale for developing and validating the natural language processing system, including references to existing systems. | 1,2,3 |
|  | 1. Specify the objectives, including whether the study describes the development or validation of the model or both. | 2,3,5-10 |
| **Methods** |  |  |
| Source of data | 1. Describe the study design or source of data (e.g. internal or external datasets) and cohort characteristics for the training, development, and validation datasets, if applicable | 3,4 |
|  | 1. Specify key elements of the study setting (e.g. primary care, secondary care, general population) including number and location of centers. | - |
|  | 1. Describe what types of texts were included and why. | 3,4 |
| Outcome | 1. Clearly define the outcome determined by the system. | 5-10 |
|  | 1. Clearly define the reference standard (e.g. manual review of notes, ICD codes). | - |
| Statistical analysis methods | 1. Specify the type of system, including whether it was self-developed or an existing system, all system-building procedures, and method for internal validation. | - |
|  | 1. Specify all measures used to assess system performance and, if relevant, to compare multiple systems. Require characteristics include the training population, whether cross-validation was employed, the testing population, and whether a completely orthogonal cohort (for example, a different hospital) was used as an independent test. | 6,7 |
| Development vs. validation | 1. For validation, identify any differences from the development data in setting, eligibility criteria, or outcome. | - |
| **Result** |  |  |
| System development | 1. Specify the number of included texts in each analysis. | 3,4 |
| System performance | 1. Report performance measures. If the approach is predictive, accuracy, area under the curve, sensitivity, and specificity should be provided along with confidence intervals if relevant. | 7,8 |
| System update | 1. Perform an error analysis. Describe any system updating arising from the validation, if done. | - |
| **Discussion** |  |  |
| Limitations | 1. Discuss any limitations of the study (such as biased sample or lack of external validation). | 10 |
| Interpretation | 1. For validation, discuss the results with reference to performance in the development data, or any other validation data. | - |
|  | 1. Give an overall interpretation of results, considering objectives, limitations, results from similar studies, and other relevant evidence. | 10,11 |
| Implications | 1. Discuss the potential clinical use of the system and implications for future research. | 9,10 |
| **Other information** |  |  |
| Supplementary information | 1. Provide information about the availability of supplementary resources, such as study protocol, datasets, and source code. | - |
| Funding | 1. Give the source of funding and the role of the funders for the present study. | - |
| **Total Score: 11** |  |  |

Zhao et al _3. (2021). Bayesian Optimization with Tree Ensembles to Improve Depression Screening on Textual Datasets.

| **Item** | **Section / Topic** | **Page NO.** |
| --- | --- | --- |
| **Title and Abstract** |  |  |
| Title | 1. Identify the study as developing and/or validating a natural language processing system, the target population, and the primary outcome. | - |
| Abstract | 1. Provide a summary of objectives, study design, setting, participants, sample size, outcome, statistical analysis, results, and conclusions | - |
| **Introduction** |  |  |
| Background and objectives | 1. Explain the medical context (including whether diagnostic or prognostic) and rationale for developing and validating the natural language processing system, including references to existing systems. | 1,2,3 |
|  | 1. Specify the objectives, including whether the study describes the development or validation of the model or both. | 2,3,5-10 |
| **Methods** |  |  |
| Source of data | 1. Describe the study design or source of data (e.g. internal or external datasets) and cohort characteristics for the training, development, and validation datasets, if applicable | 3,4 |
|  | 1. Specify key elements of the study setting (e.g. primary care, secondary care, general population) including number and location of centers. | - |
|  | 1. Describe what types of texts were included and why. | 3,4 |
| Outcome | 1. Clearly define the outcome determined by the system. | 5-10 |
|  | 1. Clearly define the reference standard (e.g. manual review of notes, ICD codes). | - |
| Statistical analysis methods | 1. Specify the type of system, including whether it was self-developed or an existing system, all system-building procedures, and method for internal validation. | - |
|  | 1. Specify all measures used to assess system performance and, if relevant, to compare multiple systems. Require characteristics include the training population, whether cross-validation was employed, the testing population, and whether a completely orthogonal cohort (for example, a different hospital) was used as an independent test. | 6,7 |
| Development vs. validation | 1. For validation, identify any differences from the development data in setting, eligibility criteria, or outcome. | - |
| **Result** |  |  |
| System development | 1. Specify the number of included texts in each analysis. | 3,4 |
| System performance | 1. Report performance measures. If the approach is predictive, accuracy, area under the curve, sensitivity, and specificity should be provided along with confidence intervals if relevant. | 7,8 |
| System update | 1. Perform an error analysis. Describe any system updating arising from the validation, if done. | - |
| **Discussion** |  |  |
| Limitations | 1. Discuss any limitations of the study (such as biased sample or lack of external validation). | 10 |
| Interpretation | 1. For validation, discuss the results with reference to performance in the development data, or any other validation data. | - |
|  | 1. Give an overall interpretation of results, considering objectives, limitations, results from similar studies, and other relevant evidence. | 10,11 |
| Implications | 1. Discuss the potential clinical use of the system and implications for future research. | 9,10 |
| **Other information** |  |  |
| Supplementary information | 1. Provide information about the availability of supplementary resources, such as study protocol, datasets, and source code. | - |
| Funding | 1. Give the source of funding and the role of the funders for the present study. | - |
| **Total Score: 11** |  |  |

Shin et al. (2022). Detection of Depression and Suicide Risk Based on Text From Clinical Interviews Using Machine Learning: Possibility of a New Objective Diagnostic Marker.

| **Item** | **Section / Topic** | **Page NO.** |
| --- | --- | --- |
| **Title and Abstract** |  |  |
| Title | 1. Identify the study as developing and/or validating a natural language processing system, the target population, and the primary outcome. | - |
| Abstract | 1. Provide a summary of objectives, study design, setting, participants, sample size, outcome, statistical analysis, results, and conclusions | - |
| **Introduction** |  |  |
| Background and objectives | 1. Explain the medical context (including whether diagnostic or prognostic) and rationale for developing and validating the natural language processing system, including references to existing systems. | 1,2 |
|  | 1. Specify the objectives, including whether the study describes the development or validation of the model or both. | 2 |
| **Methods** |  |  |
| Source of data | 1. Describe the study design or source of data (e.g. internal or external datasets) and cohort characteristics for the training, development, and validation datasets, if applicable | 2-5 |
|  | 1. Specify key elements of the study setting (e.g. primary care, secondary care, general population) including number and location of centers. | - |
|  | 1. Describe what types of texts were included and why. | 2 |
| Outcome | 1. Clearly define the outcome determined by the system. | 4-6 |
|  | 1. Clearly define the reference standard (e.g. manual review of notes, ICD codes). | 3 |
| Statistical analysis methods | 1. Specify the type of system, including whether it was self-developed or an existing system, all system-building procedures, and method for internal validation. | - |
|  | 1. Specify all measures used to assess system performance and, if relevant, to compare multiple systems. Require characteristics include the training population, whether cross-validation was employed, the testing population, and whether a completely orthogonal cohort (for example, a different hospital) was used as an independent test. | 4,5 |
| Development vs. validation | 1. For validation, identify any differences from the development data in setting, eligibility criteria, or outcome. | - |
| **Result** |  |  |
| System development | 1. Specify the number of included texts in each analysis. | 4 |
| System performance | 1. Report performance measures. If the approach is predictive, accuracy, area under the curve, sensitivity, and specificity should be provided along with confidence intervals if relevant. | 6,7 |
| System update | 1. Perform an error analysis. Describe any system updating arising from the validation, if done. | - |
| **Discussion** |  |  |
| Limitations | 1. Discuss any limitations of the study (such as biased sample or lack of external validation). | 9 |
| Interpretation | 1. For validation, discuss the results with reference to performance in the development data, or any other validation data. | 4-7 |
|  | 1. Give an overall interpretation of results, considering objectives, limitations, results from similar studies, and other relevant evidence. | 7-9 |
| Implications | 1. Discuss the potential clinical use of the system and implications for future research. | 7,8,12 |
| **Other information** |  |  |
| Supplementary information | 1. Provide information about the availability of supplementary resources, such as study protocol, datasets, and source code. | - |
| Funding | 1. Give the source of funding and the role of the funders for the present study. | 10 |
| **Total Score: 14** |  |  |

Cariola et al. (2022). Language Use in Mother-Adolescent Dyadic Interaction: Preliminary Results.

| **Item** | **Section / Topic** | **Page NO.** |
| --- | --- | --- |
| **Title and Abstract** |  |  |
| Title | 1. Identify the study as developing and/or validating a natural language processing system, the target population, and the primary outcome. | - |
| Abstract | 1. Provide a summary of objectives, study design, setting, participants, sample size, outcome, statistical analysis, results, and conclusions | 0 |
| **Introduction** |  |  |
| Background and objectives | 1. Explain the medical context (including whether diagnostic or prognostic) and rationale for developing and validating the natural language processing system, including references to existing systems. | 1,2,3 |
|  | 1. Specify the objectives, including whether the study describes the development or validation of the model or both. | 2,3,4 |
| **Methods** |  |  |
| Source of data | 1. Describe the study design or source of data (e.g. internal or external datasets) and cohort characteristics for the training, development, and validation datasets, if applicable | 2,3,4 |
|  | 1. Specify key elements of the study setting (e.g. primary care, secondary care, general population) including number and location of centers. | - |
|  | 1. Describe what types of texts were included and why. | 3,4 |
| Outcome | 1. Clearly define the outcome determined by the system. | 4,5 |
|  | 1. Clearly define the reference standard (e.g. manual review of notes, ICD codes). | - |
| Statistical analysis methods | 1. Specify the type of system, including whether it was self-developed or an existing system, all system-building procedures, and method for internal validation. | 3,4 |
|  | 1. Specify all measures used to assess system performance and, if relevant, to compare multiple systems. Require characteristics include the training population, whether cross-validation was employed, the testing population, and whether a completely orthogonal cohort (for example, a different hospital) was used as an independent test. | - |
| Development vs. validation | 1. For validation, identify any differences from the development data in setting, eligibility criteria, or outcome. | - |
| **Result** |  |  |
| System development | 1. Specify the number of included texts in each analysis. | - |
| System performance | 1. Report performance measures. If the approach is predictive, accuracy, area under the curve, sensitivity, and specificity should be provided along with confidence intervals if relevant. | 4,5 |
| System update | 1. Perform an error analysis. Describe any system updating arising from the validation, if done. | - |
| **Discussion** |  |  |
| Limitations | 1. Discuss any limitations of the study (such as biased sample or lack of external validation). | - |
| Interpretation | 1. For validation, discuss the results with reference to performance in the development data, or any other validation data. | - |
|  | 1. Give an overall interpretation of results, considering objectives, limitations, results from similar studies, and other relevant evidence. | 5,6 |
| Implications | 1. Discuss the potential clinical use of the system and implications for future research. | - |
| **Other information** |  |  |
| Supplementary information | 1. Provide information about the availability of supplementary resources, such as study protocol, datasets, and source code. | - |
| Funding | 1. Give the source of funding and the role of the funders for the present study. | 1 |
| **Total Score: 10** |  |  |

Munthuli et al _1. (2023). Classification and Analysis of Text Transcription from Thai Depression Assessment Tasks among Patients with Depression.

| **Item** | **Section / Topic** | **Page NO.** |
| --- | --- | --- |
| **Title and Abstract** |  |  |
| Title | 1. Identify the study as developing and/or validating a natural language processing system, the target population, and the primary outcome. | - |
| Abstract | 1. Provide a summary of objectives, study design, setting, participants, sample size, outcome, statistical analysis, results, and conclusions | 1 |
| **Introduction** |  |  |
| Background and objectives | 1. Explain the medical context (including whether diagnostic or prognostic) and rationale for developing and validating the natural language processing system, including references to existing systems. | 2,3 |
|  | 1. Specify the objectives, including whether the study describes the development or validation of the model or both. | 3 |
| **Methods** |  |  |
| Source of data | 1. Describe the study design or source of data (e.g. internal or external datasets) and cohort characteristics for the training, development, and validation datasets, if applicable | 5,6 |
|  | 1. Specify key elements of the study setting (e.g. primary care, secondary care, general population) including number and location of centers. | - |
|  | 1. Describe what types of texts were included and why. | 2,3,5,6,7 |
| Outcome | 1. Clearly define the outcome determined by the system. | 9 - 14 |
|  | 1. Clearly define the reference standard (e.g. manual review of notes, ICD codes). | - |
| Statistical analysis methods | 1. Specify the type of system, including whether it was self-developed or an existing system, all system-building procedures, and method for internal validation. | 7 |
|  | 1. Specify all measures used to assess system performance and, if relevant, to compare multiple systems. Require characteristics include the training population, whether cross-validation was employed, the testing population, and whether a completely orthogonal cohort (for example, a different hospital) was used as an independent test. | 7,8,9 |
| Development vs. validation | 1. For validation, identify any differences from the development data in setting, eligibility criteria, or outcome. | - |
| **Result** |  |  |
| System development | 1. Specify the number of included texts in each analysis. | - |
| System performance | 1. Report performance measures. If the approach is predictive, accuracy, area under the curve, sensitivity, and specificity should be provided along with confidence intervals if relevant. | 8,9,10 |
| System update | 1. Perform an error analysis. Describe any system updating arising from the validation, if done. | - |
| **Discussion** |  |  |
| Limitations | 1. Discuss any limitations of the study (such as biased sample or lack of external validation). | 13,14 |
| Interpretation | 1. For validation, discuss the results with reference to performance in the development data, or any other validation data. | 9,12 |
|  | 1. Give an overall interpretation of results, considering objectives, limitations, results from similar studies, and other relevant evidence. | 12-19 |
| Implications | 1. Discuss the potential clinical use of the system and implications for future research. | 12,13,14 |
| **Other information** |  |  |
| Supplementary information | 1. Provide information about the availability of supplementary resources, such as study protocol, datasets, and source code. | 19 |
| Funding | 1. Give the source of funding and the role of the funders for the present study. | 2 |
| **Total Score: 15** |  |  |

Munthuli et al _2. (2023). Classification and Analysis of Text Transcription from Thai Depression Assessment Tasks among Patients with Depression.

| **Item** | **Section / Topic** | **Page NO.** |
| --- | --- | --- |
| **Title and Abstract** |  |  |
| Title | 1. Identify the study as developing and/or validating a natural language processing system, the target population, and the primary outcome. | - |
| Abstract | 1. Provide a summary of objectives, study design, setting, participants, sample size, outcome, statistical analysis, results, and conclusions | 1 |
| **Introduction** |  |  |
| Background and objectives | 1. Explain the medical context (including whether diagnostic or prognostic) and rationale for developing and validating the natural language processing system, including references to existing systems. | 2,3 |
|  | 1. Specify the objectives, including whether the study describes the development or validation of the model or both. | 3 |
| **Methods** |  |  |
| Source of data | 1. Describe the study design or source of data (e.g. internal or external datasets) and cohort characteristics for the training, development, and validation datasets, if applicable | 5,6 |
|  | 1. Specify key elements of the study setting (e.g. primary care, secondary care, general population) including number and location of centers. | - |
|  | 1. Describe what types of texts were included and why. | 2,3,5,6,7 |
| Outcome | 1. Clearly define the outcome determined by the system. | 9 - 14 |
|  | 1. Clearly define the reference standard (e.g. manual review of notes, ICD codes). | - |
| Statistical analysis methods | 1. Specify the type of system, including whether it was self-developed or an existing system, all system-building procedures, and method for internal validation. | 7 |
|  | 1. Specify all measures used to assess system performance and, if relevant, to compare multiple systems. Require characteristics include the training population, whether cross-validation was employed, the testing population, and whether a completely orthogonal cohort (for example, a different hospital) was used as an independent test. | 7,8,9 |
| Development vs. validation | 1. For validation, identify any differences from the development data in setting, eligibility criteria, or outcome. | - |
| **Result** |  |  |
| System development | 1. Specify the number of included texts in each analysis. | - |
| System performance | 1. Report performance measures. If the approach is predictive, accuracy, area under the curve, sensitivity, and specificity should be provided along with confidence intervals if relevant. | 8,9,10 |
| System update | 1. Perform an error analysis. Describe any system updating arising from the validation, if done. | - |
| **Discussion** |  |  |
| Limitations | 1. Discuss any limitations of the study (such as biased sample or lack of external validation). | 13,14 |
| Interpretation | 1. For validation, discuss the results with reference to performance in the development data, or any other validation data. | 9,12 |
|  | 1. Give an overall interpretation of results, considering objectives, limitations, results from similar studies, and other relevant evidence. | 12-19 |
| Implications | 1. Discuss the potential clinical use of the system and implications for future research. | 12,13,14 |
| **Other information** |  |  |
| Supplementary information | 1. Provide information about the availability of supplementary resources, such as study protocol, datasets, and source code. | 19 |
| Funding | 1. Give the source of funding and the role of the funders for the present study. | 2 |
| **Total Score: 15** |  |  |

Munthuli et al _3. (2023). Classification and Analysis of Text Transcription from Thai Depression Assessment Tasks among Patients with Depression.

| **Item** | **Section / Topic** | **Page NO.** |
| --- | --- | --- |
| **Title and Abstract** |  |  |
| Title | 1. Identify the study as developing and/or validating a natural language processing system, the target population, and the primary outcome. | - |
| Abstract | 1. Provide a summary of objectives, study design, setting, participants, sample size, outcome, statistical analysis, results, and conclusions | 1 |
| **Introduction** |  |  |
| Background and objectives | 1. Explain the medical context (including whether diagnostic or prognostic) and rationale for developing and validating the natural language processing system, including references to existing systems. | 2,3 |
|  | 1. Specify the objectives, including whether the study describes the development or validation of the model or both. | 3 |
| **Methods** |  |  |
| Source of data | 1. Describe the study design or source of data (e.g. internal or external datasets) and cohort characteristics for the training, development, and validation datasets, if applicable | 5,6 |
|  | 1. Specify key elements of the study setting (e.g. primary care, secondary care, general population) including number and location of centers. | - |
|  | 1. Describe what types of texts were included and why. | 2,3,5,6,7 |
| Outcome | 1. Clearly define the outcome determined by the system. | 9 - 14 |
|  | 1. Clearly define the reference standard (e.g. manual review of notes, ICD codes). | - |
| Statistical analysis methods | 1. Specify the type of system, including whether it was self-developed or an existing system, all system-building procedures, and method for internal validation. | 7 |
|  | 1. Specify all measures used to assess system performance and, if relevant, to compare multiple systems. Require characteristics include the training population, whether cross-validation was employed, the testing population, and whether a completely orthogonal cohort (for example, a different hospital) was used as an independent test. | 7,8,9 |
| Development vs. validation | 1. For validation, identify any differences from the development data in setting, eligibility criteria, or outcome. | - |
| **Result** |  |  |
| System development | 1. Specify the number of included texts in each analysis. | - |
| System performance | 1. Report performance measures. If the approach is predictive, accuracy, area under the curve, sensitivity, and specificity should be provided along with confidence intervals if relevant. | 8,9,10 |
| System update | 1. Perform an error analysis. Describe any system updating arising from the validation, if done. | - |
| **Discussion** |  |  |
| Limitations | 1. Discuss any limitations of the study (such as biased sample or lack of external validation). | 13,14 |
| Interpretation | 1. For validation, discuss the results with reference to performance in the development data, or any other validation data. | 9,12 |
|  | 1. Give an overall interpretation of results, considering objectives, limitations, results from similar studies, and other relevant evidence. | 12-19 |
| Implications | 1. Discuss the potential clinical use of the system and implications for future research. | 12,13,14 |
| **Other information** |  |  |
| Supplementary information | 1. Provide information about the availability of supplementary resources, such as study protocol, datasets, and source code. | 19 |
| Funding | 1. Give the source of funding and the role of the funders for the present study. | 2 |
| **Total Score: 15** |  |  |

Tlachac et al. (2023). Automated Construction of Lexicons to Improve Depression Screening With Text Messages.

| **Item** | **Section / Topic** | **Page NO.** |
| --- | --- | --- |
| **Title and Abstract** |  |  |
| Title | 1. Identify the study as developing and/or validating a natural language processing system, the target population, and the primary outcome. | - |
| Abstract | 1. Provide a summary of objectives, study design, setting, participants, sample size, outcome, statistical analysis, results, and conclusions | - |
| **Introduction** |  |  |
| Background and objectives | 1. Explain the medical context (including whether diagnostic or prognostic) and rationale for developing and validating the natural language processing system, including references to existing systems. | 2751,2752 |
|  | 1. Specify the objectives, including whether the study describes the development or validation of the model or both. | 2751-2753 |
| **Methods** |  |  |
| Source of data | 1. Describe the study design or source of data (e.g. internal or external datasets) and cohort characteristics for the training, development, and validation datasets, if applicable | 2753 |
|  | 1. Specify key elements of the study setting (e.g. primary care, secondary care, general population) including number and location of centers. | - |
|  | 1. Describe what types of texts were included and why. | 2753 |
| Outcome | 1. Clearly define the outcome determined by the system. | 2755-2757 |
|  | 1. Clearly define the reference standard (e.g. manual review of notes, ICD codes). | - |
| Statistical analysis methods | 1. Specify the type of system, including whether it was self-developed or an existing system, all system-building procedures, and method for internal validation. | - |
|  | 1. Specify all measures used to assess system performance and, if relevant, to compare multiple systems. Require characteristics include the training population, whether cross-validation was employed, the testing population, and whether a completely orthogonal cohort (for example, a different hospital) was used as an independent test. | 2755,2756 |
| Development vs. validation | 1. For validation, identify any differences from the development data in setting, eligibility criteria, or outcome. | - |
| **Result** |  |  |
| System development | 1. Specify the number of included texts in each analysis. | 2753 |
| System performance | 1. Report performance measures. If the approach is predictive, accuracy, area under the curve, sensitivity, and specificity should be provided along with confidence intervals if relevant. | 2755-2757 |
| System update | 1. Perform an error analysis. Describe any system updating arising from the validation, if done. | - |
| **Discussion** |  |  |
| Limitations | 1. Discuss any limitations of the study (such as biased sample or lack of external validation). | 2758 |
| Interpretation | 1. For validation, discuss the results with reference to performance in the development data, or any other validation data. | - |
|  | 1. Give an overall interpretation of results, considering objectives, limitations, results from similar studies, and other relevant evidence. | 2758,2759 |
| Implications | 1. Discuss the potential clinical use of the system and implications for future research. | 2758,2759 |
| **Other information** |  |  |
| Supplementary information | 1. Provide information about the availability of supplementary resources, such as study protocol, datasets, and source code. | 2755 |
| Funding | 1. Give the source of funding and the role of the funders for the present study. | 2751 |
| **Total Score: 13** |  |  |

Jihoon et al. (2024). Development of Depression Detection Algorithm Using Text Scripts of Routine Psychiatric Interview.

| **Item** | **Section / Topic** | **Page NO.** |
| --- | --- | --- |
| **Title and Abstract** |  |  |
| Title | 1. Identify the study as developing and/or validating a natural language processing system, the target population, and the primary outcome. | - |
| Abstract | 1. Provide a summary of objectives, study design, setting, participants, sample size, outcome, statistical analysis, results, and conclusions | - |
| **Introduction** |  |  |
| Background and objectives | 1. Explain the medical context (including whether diagnostic or prognostic) and rationale for developing and validating the natural language processing system, including references to existing systems. | 2 |
|  | 1. Specify the objectives, including whether the study describes the development or validation of the model or both. | 1 |
| **Methods** |  |  |
| Source of data | 1. Describe the study design or source of data (e.g. internal or external datasets) and cohort characteristics for the training, development, and validation datasets, if applicable | 3,4,5 |
|  | 1. Specify key elements of the study setting (e.g. primary care, secondary care, general population) including number and location of centers. | 3 |
|  | 1. Describe what types of texts were included and why. | 3 |
| Outcome | 1. Clearly define the outcome determined by the system. | 5 |
|  | 1. Clearly define the reference standard (e.g. manual review of notes, ICD codes). | 3,4 |
| Statistical analysis methods | 1. Specify the type of system, including whether it was self-developed or an existing system, all system-building procedures, and method for internal validation. | 3 |
|  | 1. Specify all measures used to assess system performance and, if relevant, to compare multiple systems. Require characteristics include the training population, whether cross-validation was employed, the testing population, and whether a completely orthogonal cohort (for example, a different hospital) was used as an independent test. | 3 |
| Development vs. validation | 1. For validation, identify any differences from the development data in setting, eligibility criteria, or outcome. | - |
| **Result** |  |  |
| System development | 1. Specify the number of included texts in each analysis. | 3,4 |
| System performance | 1. Report performance measures. If the approach is predictive, accuracy, area under the curve, sensitivity, and specificity should be provided along with confidence intervals if relevant. | 5,6 |
| System update | 1. Perform an error analysis. Describe any system updating arising from the validation, if done. | - |
| **Discussion** |  |  |
| Limitations | 1. Discuss any limitations of the study (such as biased sample or lack of external validation). | 6,7 |
| Interpretation | 1. For validation, discuss the results with reference to performance in the development data, or any other validation data. | 5,6 |
|  | 1. Give an overall interpretation of results, considering objectives, limitations, results from similar studies, and other relevant evidence. | 5,6,7 |
| Implications | 1. Discuss the potential clinical use of the system and implications for future research. | 6,7 |
| **Other information** |  |  |
| Supplementary information | 1. Provide information about the availability of supplementary resources, such as study protocol, datasets, and source code. | 7 |
| Funding | 1. Give the source of funding and the role of the funders for the present study. | 7 |
| **Total Score: 17** |  |  |

Shin et al. (2024). Using Large Language Models to Detect Depression From User-Generated Diary Text Data as a Novel Approach in Digital Mental Health Screening: Instrument Validation Study.

| **Item** | **Section / Topic** | **Page NO.** |
| --- | --- | --- |
| **Title and Abstract** |  |  |
| Title | 1. Identify the study as developing and/or validating a natural language processing system, the target population, and the primary outcome. | - |
| Abstract | 1. Provide a summary of objectives, study design, setting, participants, sample size, outcome, statistical analysis, results, and conclusions | - |
| **Introduction** |  |  |
| Background and objectives | 1. Explain the medical context (including whether diagnostic or prognostic) and rationale for developing and validating the natural language processing system, including references to existing systems. | 1,2 |
|  | 1. Specify the objectives, including whether the study describes the development or validation of the model or both. | 2 |
| **Methods** |  |  |
| Source of data | 1. Describe the study design or source of data (e.g. internal or external datasets) and cohort characteristics for the training, development, and validation datasets, if applicable | 2-5 |
|  | 1. Specify key elements of the study setting (e.g. primary care, secondary care, general population) including number and location of centers. | - |
|  | 1. Describe what types of texts were included and why. | 2 |
| Outcome | 1. Clearly define the outcome determined by the system. | 4-6 |
|  | 1. Clearly define the reference standard (e.g. manual review of notes, ICD codes). | 3 |
| Statistical analysis methods | 1. Specify the type of system, including whether it was self-developed or an existing system, all system-building procedures, and method for internal validation. | - |
|  | 1. Specify all measures used to assess system performance and, if relevant, to compare multiple systems. Require characteristics include the training population, whether cross-validation was employed, the testing population, and whether a completely orthogonal cohort (for example, a different hospital) was used as an independent test. | 4,5 |
| Development vs. validation | 1. For validation, identify any differences from the development data in setting, eligibility criteria, or outcome. | - |
| **Result** |  |  |
| System development | 1. Specify the number of included texts in each analysis. | 4 |
| System performance | 1. Report performance measures. If the approach is predictive, accuracy, area under the curve, sensitivity, and specificity should be provided along with confidence intervals if relevant. | 6,7 |
| System update | 1. Perform an error analysis. Describe any system updating arising from the validation, if done. | - |
| **Discussion** |  |  |
| Limitations | 1. Discuss any limitations of the study (such as biased sample or lack of external validation). | 9 |
| Interpretation | 1. For validation, discuss the results with reference to performance in the development data, or any other validation data. | 4-7 |
|  | 1. Give an overall interpretation of results, considering objectives, limitations, results from similar studies, and other relevant evidence. | 7-9 |
| Implications | 1. Discuss the potential clinical use of the system and implications for future research. | 7,8,12 |
| **Other information** |  |  |
| Supplementary information | 1. Provide information about the availability of supplementary resources, such as study protocol, datasets, and source code. | - |
| Funding | 1. Give the source of funding and the role of the funders for the present study. | 10 |
| **Total Score: 14** |  |  |

Xu et al. (2025). Deep learning-based detection of depression by fusing auditory, visual and textual clues.

| **Item** | **Section / Topic** | **Page NO.** |
| --- | --- | --- |
| **Title and Abstract** |  |  |
| Title | 1. Identify the study as developing and/or validating a natural language processing system, the target population, and the primary outcome. | 1 |
| Abstract | 1. Provide a summary of objectives, study design, setting, participants, sample size, outcome, statistical analysis, results, and conclusions | 1 |
| **Introduction** |  |  |
| Background and objectives | 1. Explain the medical context (including whether diagnostic or prognostic) and rationale for developing and validating the natural language processing system, including references to existing systems. | 1,2,3 |
|  | 1. Specify the objectives, including whether the study describes the development or validation of the model or both. | 3 |
| **Methods** |  |  |
| Source of data | 1. Describe the study design or source of data (e.g. internal or external datasets) and cohort characteristics for the training, development, and validation datasets, if applicable | 3 |
|  | 1. Specify key elements of the study setting (e.g. primary care, secondary care, general population) including number and location of centers. | 1,3 |
|  | 1. Describe what types of texts were included and why. | 2,3,4 |
| Outcome | 1. Clearly define the outcome determined by the system. | 7 |
|  | 1. Clearly define the reference standard (e.g. manual review of notes, ICD codes). | 3,4 |
| Statistical analysis methods | 1. Specify the type of system, including whether it was self-developed or an existing system, all system-building procedures, and method for internal validation. | 4,5 |
|  | 1. Specify all measures used to assess system performance and, if relevant, to compare multiple systems. Require characteristics include the training population, whether cross-validation was employed, the testing population, and whether a completely orthogonal cohort (for example, a different hospital) was used as an independent test. | 4,6,7 |
| Development vs. validation | 1. For validation, identify any differences from the development data in setting, eligibility criteria, or outcome. | 3,9 |
| **Result** |  |  |
| System development | 1. Specify the number of included texts in each analysis. | - |
| System performance | 1. Report performance measures. If the approach is predictive, accuracy, area under the curve, sensitivity, and specificity should be provided along with confidence intervals if relevant. | 6,7 |
| System update | 1. Perform an error analysis. Describe any system updating arising from the validation, if done. | - |
| **Discussion** |  |  |
| Limitations | 1. Discuss any limitations of the study (such as biased sample or lack of external validation). | 9 |
| Interpretation | 1. For validation, discuss the results with reference to performance in the development data, or any other validation data. | 9 |
|  | 1. Give an overall interpretation of results, considering objectives, limitations, results from similar studies, and other relevant evidence. | 8 |
| Implications | 1. Discuss the potential clinical use of the system and implications for future research. | 8,9 |
| **Other information** |  |  |
| Supplementary information | 1. Provide information about the availability of supplementary resources, such as study protocol, datasets, and source code. | 10 |
| Funding | 1. Give the source of funding and the role of the funders for the present study. | 10 |
| **Total Score: 19** |  |  |
