# Supplementary material for: Text-Based Depression Estimation Using Machine Learning With Standard Labels: Systematic Review and Meta-Analysis
Source: J Med Internet Res. 2026 Feb 11;28:e82686. doi: 10.2196/82686 (PMC12936666; doi:10.2196/82686)

[Supplementary Figures3. Main forest plot of pooled effect sizes using random-effects model with HKSJ. 1](#_Toc216310473)

[Supplementary Figures4. Subgroup forest plots (text representation, annotation source, model architecture, text source) generated using the R meta package. 2](#_Toc216310474)

[(A) Text representation 2](#_Toc216310475)

[(B) Label 3](#_Toc216310476)

[(C) Model architecture 4](#_Toc216310477)

[(D) Text source 5](#_Toc216310478)

[Supplementary Figures5. Bubble plots of univariable meta-regression (TRIPOD score, positive rate, log-transformed sample size) using HKSJ-adjusted models. 6](#_Toc216310479)

[(A) Regression of Fisher’s Z on TRIPOD reporting score 6](#_Toc216310480)

[(B) Regression on Positive Rate 7](#_Toc216310481)

[(C) Regression on Log-transformed Sample Size 8](#_Toc216310482)

# Supplementary Figures3. Main forest plot of pooled effect sizes using random-effects model with HKSJ.


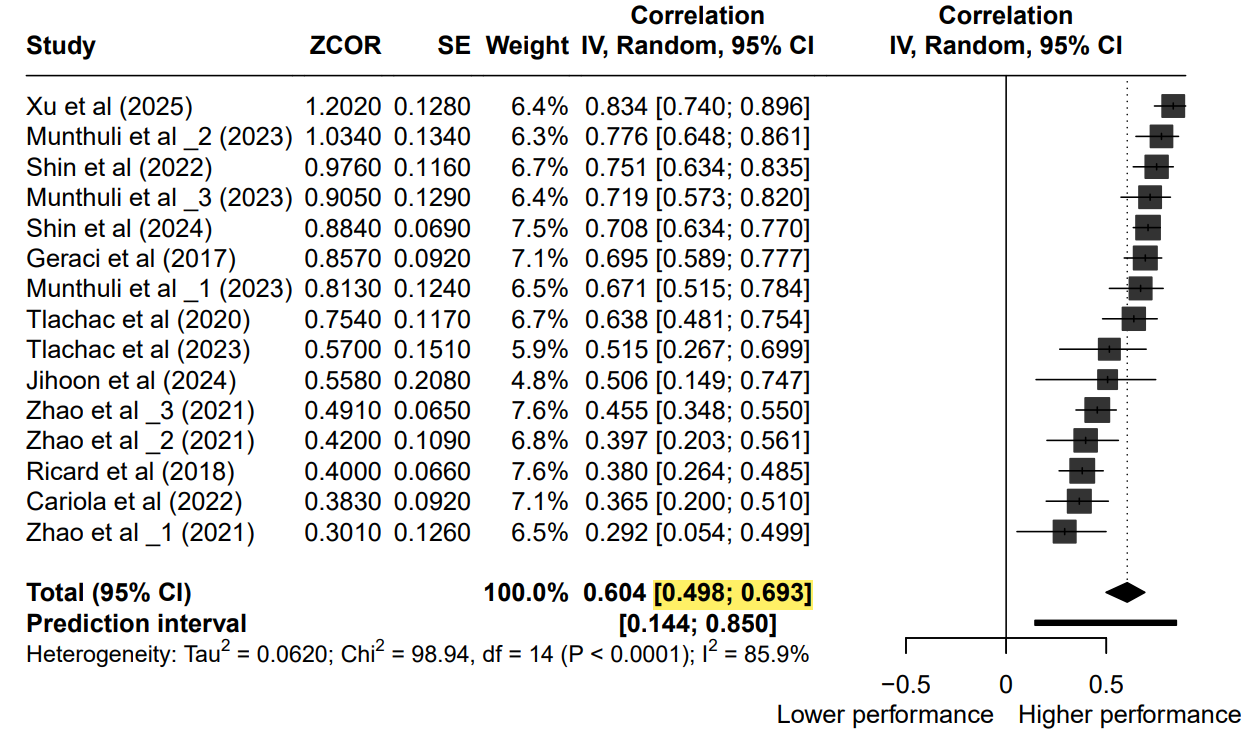


# Supplementary Figures4. Subgroup forest plots (text representation, annotation source, model architecture, text source) generated using the R meta package.

## (A) Text representation


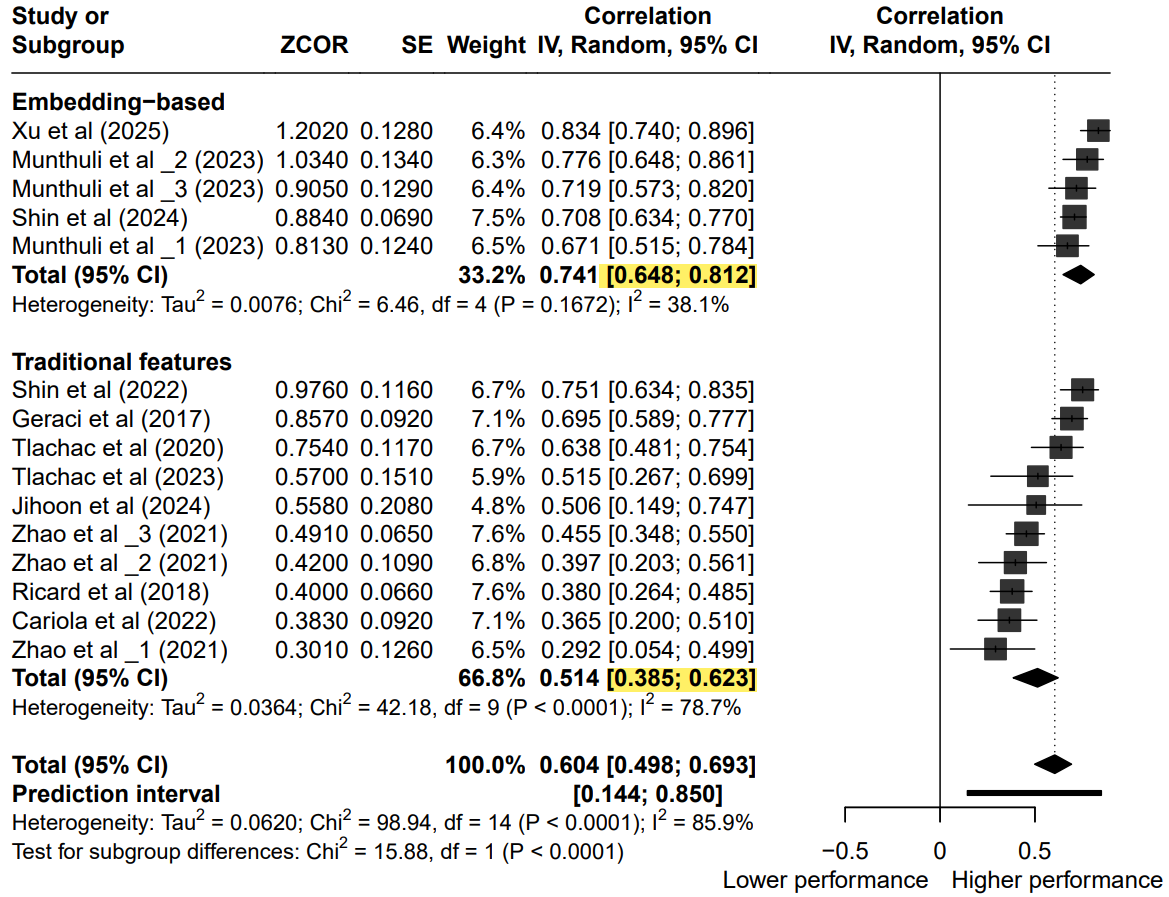


## Label


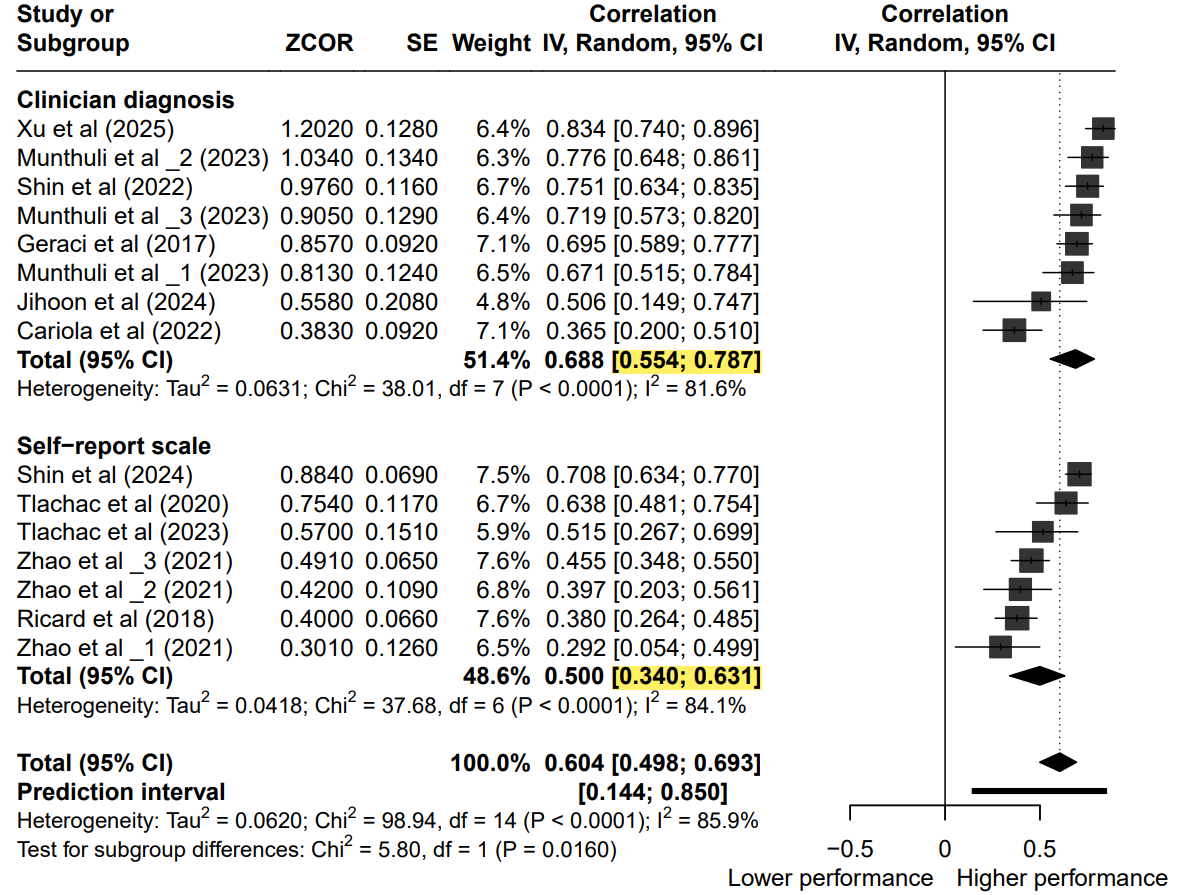


## Model architecture


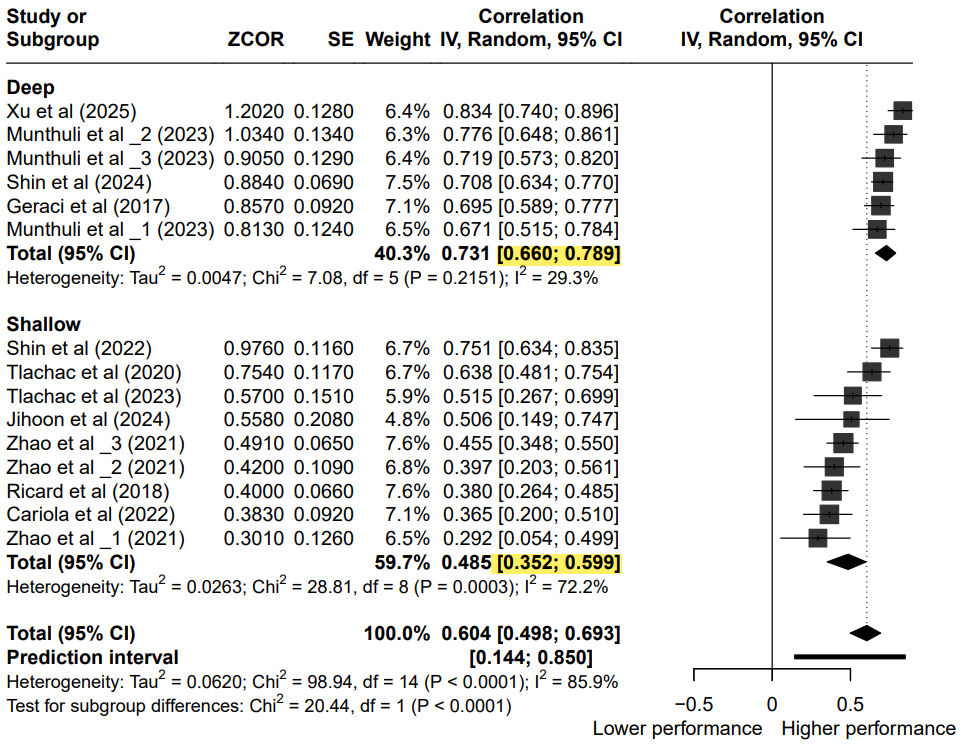


## Text source


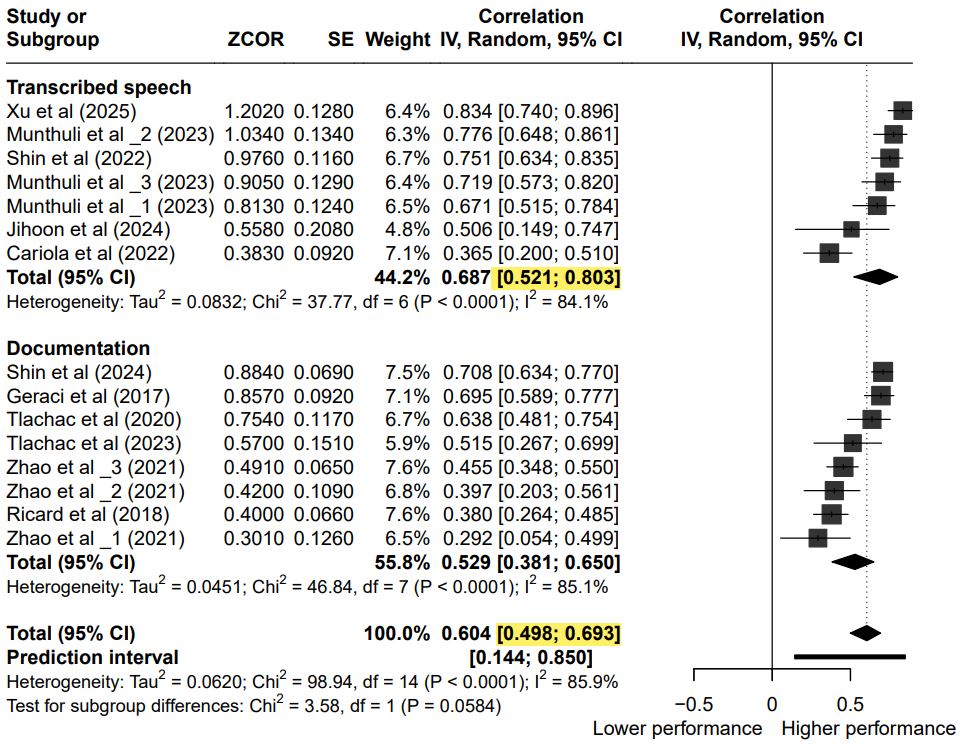


# Supplementary Figures5. Bubble plots of univariable meta-regression (TRIPOD score, positive rate, log-transformed sample size) using HKSJ-adjusted models.

## Regression of Fisher’s Z on TRIPOD reporting score


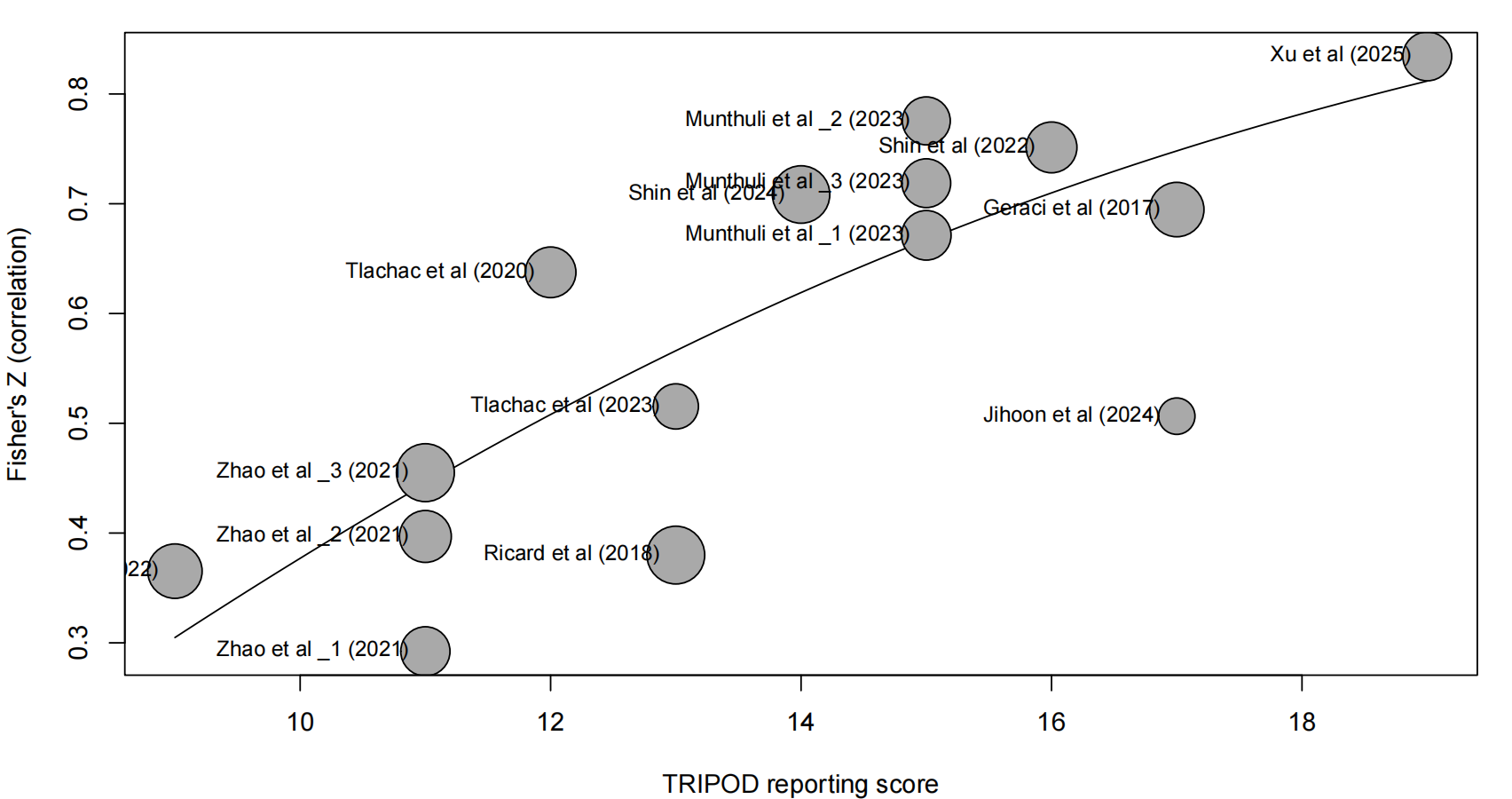


## Regression on Positive Rate


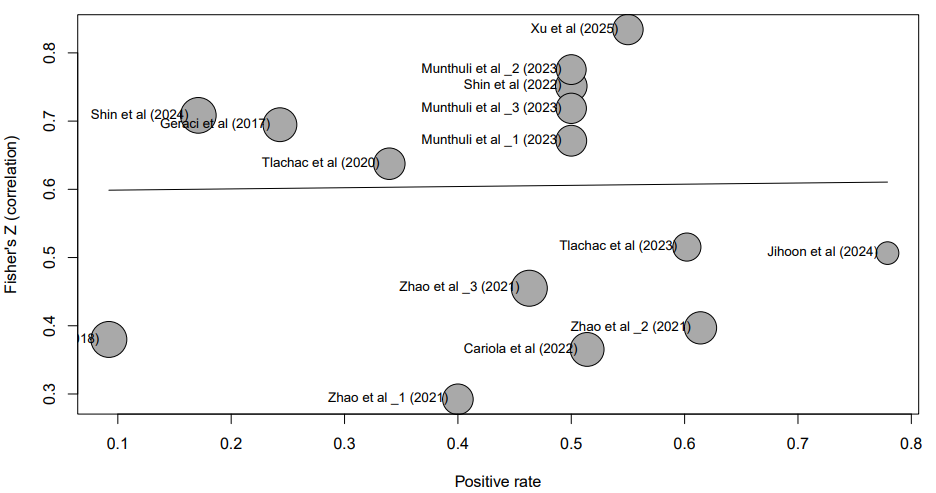


## Regression on Log-transformed Sample Size


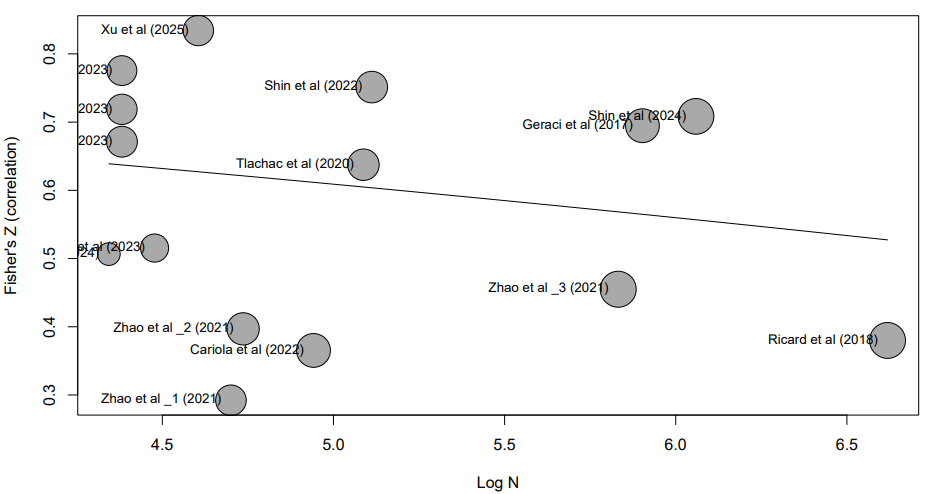

Supplement: Multimedia Appendix 5 [file jmir_v28i1e82686_app5.docx]
