# Supplementary material for: Text-Based Depression Estimation Using Machine Learning With Standard Labels: Systematic Review and Meta-Analysis
Source: J Med Internet Res. 2026 Feb 11;28:e82686. doi: 10.2196/82686 (PMC12936666; doi:10.2196/82686)

[Supplementary Figures1. Forest plots of subgroup analyses by (A) text representation, (B) annotation source, (C) model architecture, and (D) text source generated using CMA4.0. 1](#_Toc9466)

[(A) Text representation 1](#_Toc13553)

[(B) Annotation source 2](#_Toc31909)

[(C) Model architecture 3](#_Toc18604)

[(D) Text source 4](#_Toc7519)

[Supplementary Figures2. Bubble plots for univariate meta-regression analyses. (A) Regression of Fisher’s Z on TRIPOD reporting score; (B) Regression on Positive Rate; (C) Regression on Log-transformed Sample Size generated using CMA4.0. Bubble sizes are proportional to study weights. 5](#_Toc20064)

[(A) Regression of Fisher’s Z on TRIPOD reporting score 5](#_Toc25025)

[(B) Regression on Positive Rate 6](#_Toc22879)

[(C) Regression on Log-transformed Sample Size 7](#_Toc11886)

# Supplementary Figures1. Forest plots of subgroup analyses by (A) text representation, (B) annotation source, (C) model architecture, and (D) text source generated using CMA4.0.

## (A) Text representation


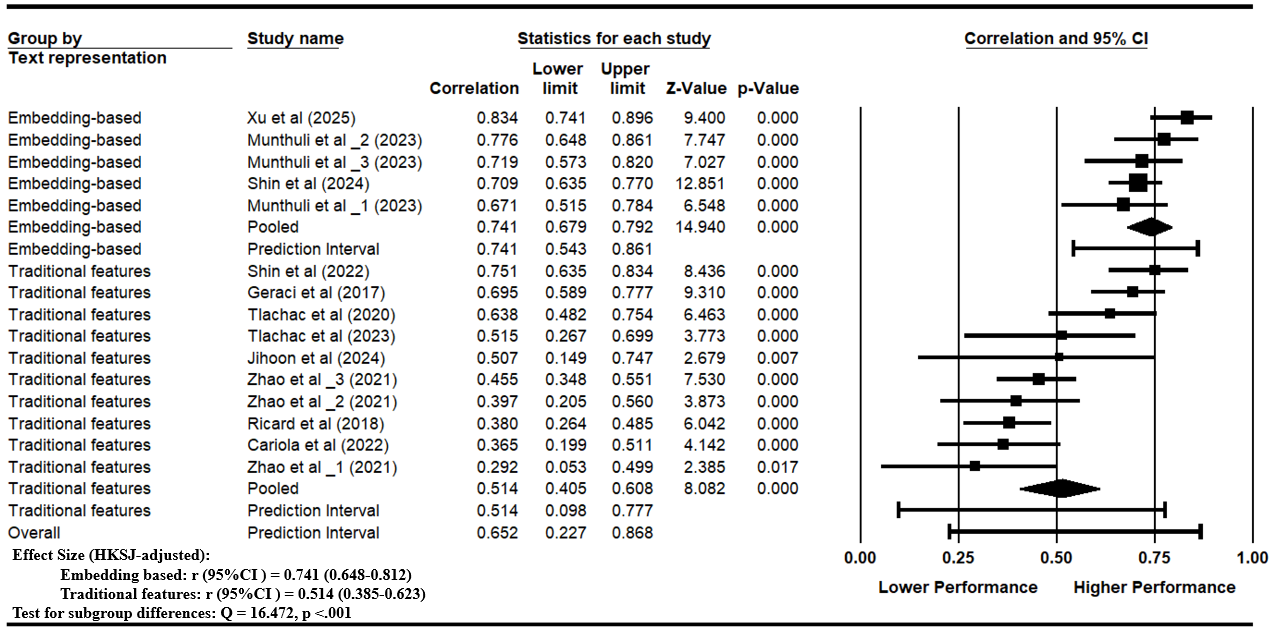


## (B) Annotation source


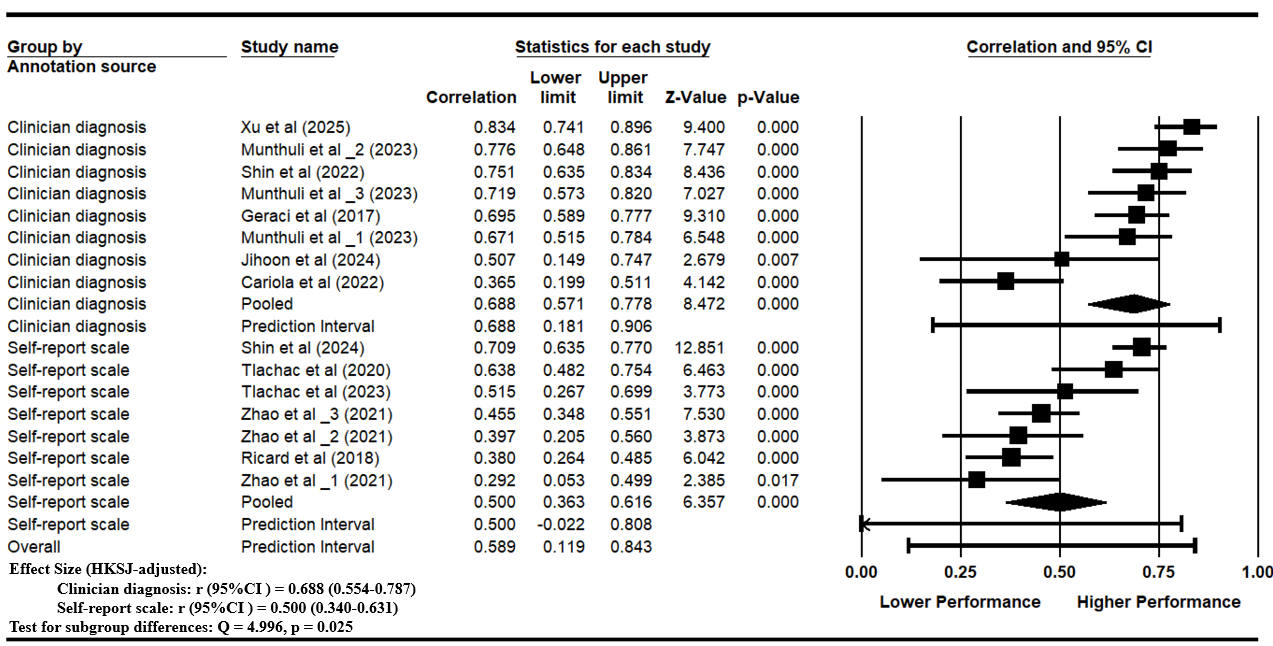


## (C) Model architecture


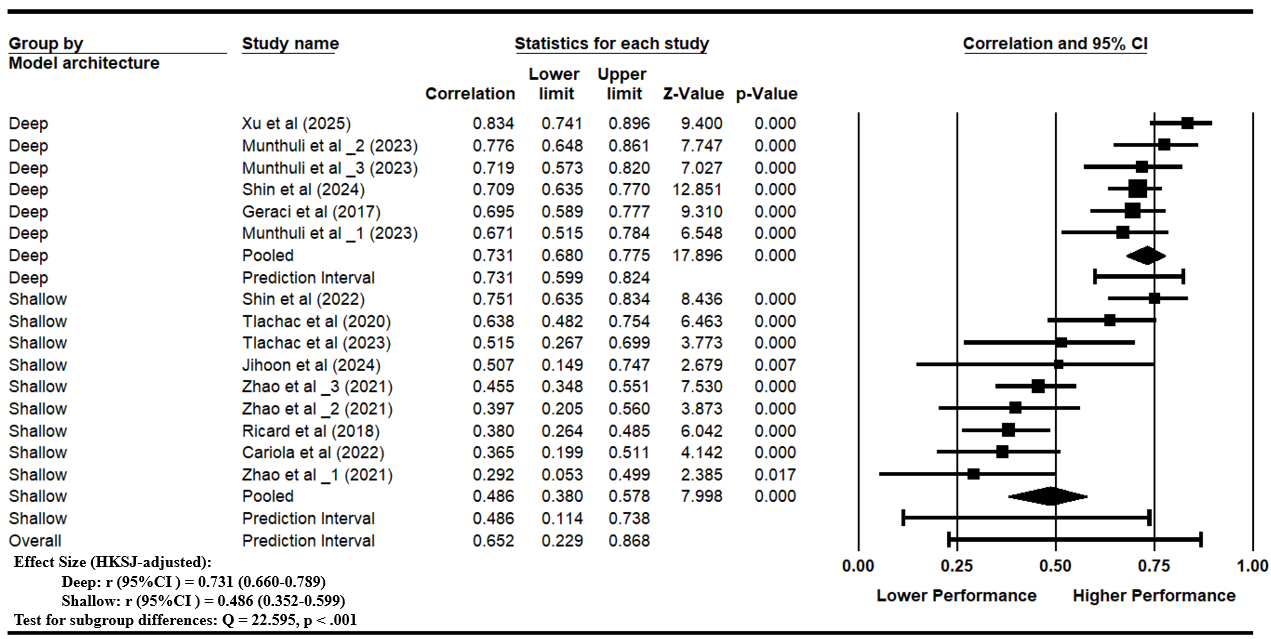


## (D) Text source


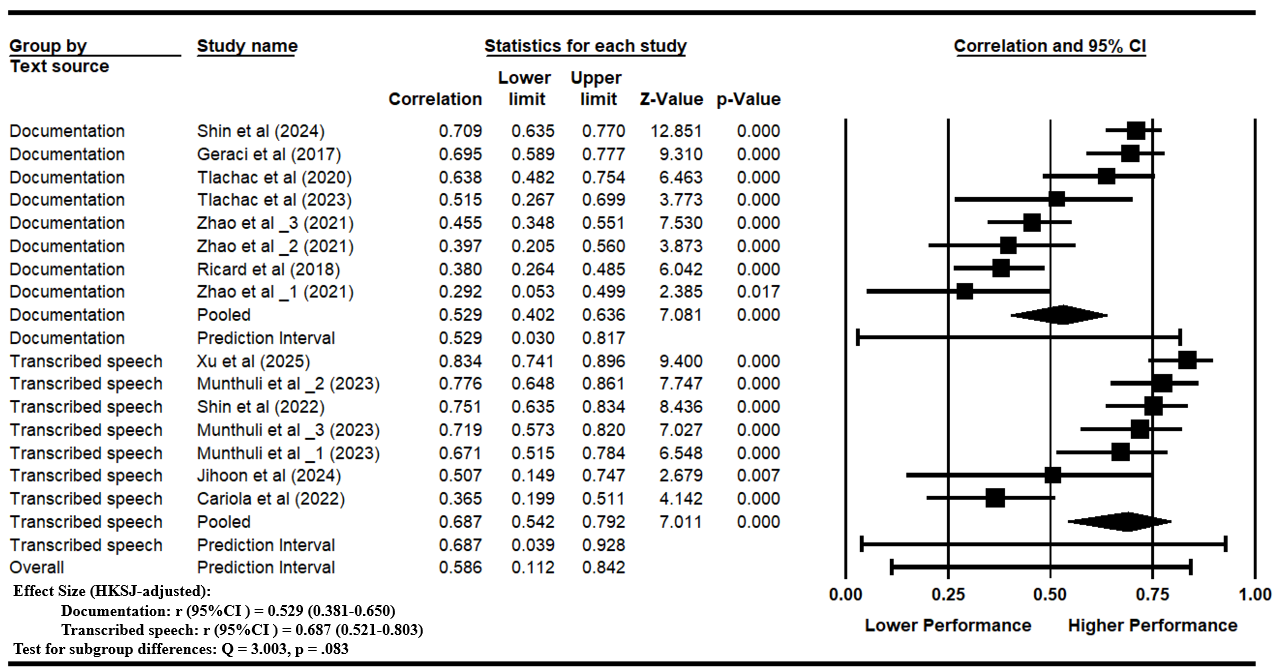


# Supplementary Figures2. Bubble plots for univariate meta-regression analyses. (A) Regression of Fisher’s Z on TRIPOD reporting score; (B) Regression on Positive Rate; (C) Regression on Log-transformed Sample Size generated using CMA4.0. Bubble sizes are proportional to study weights.

## Regression of Fisher’s Z on TRIPOD reporting score


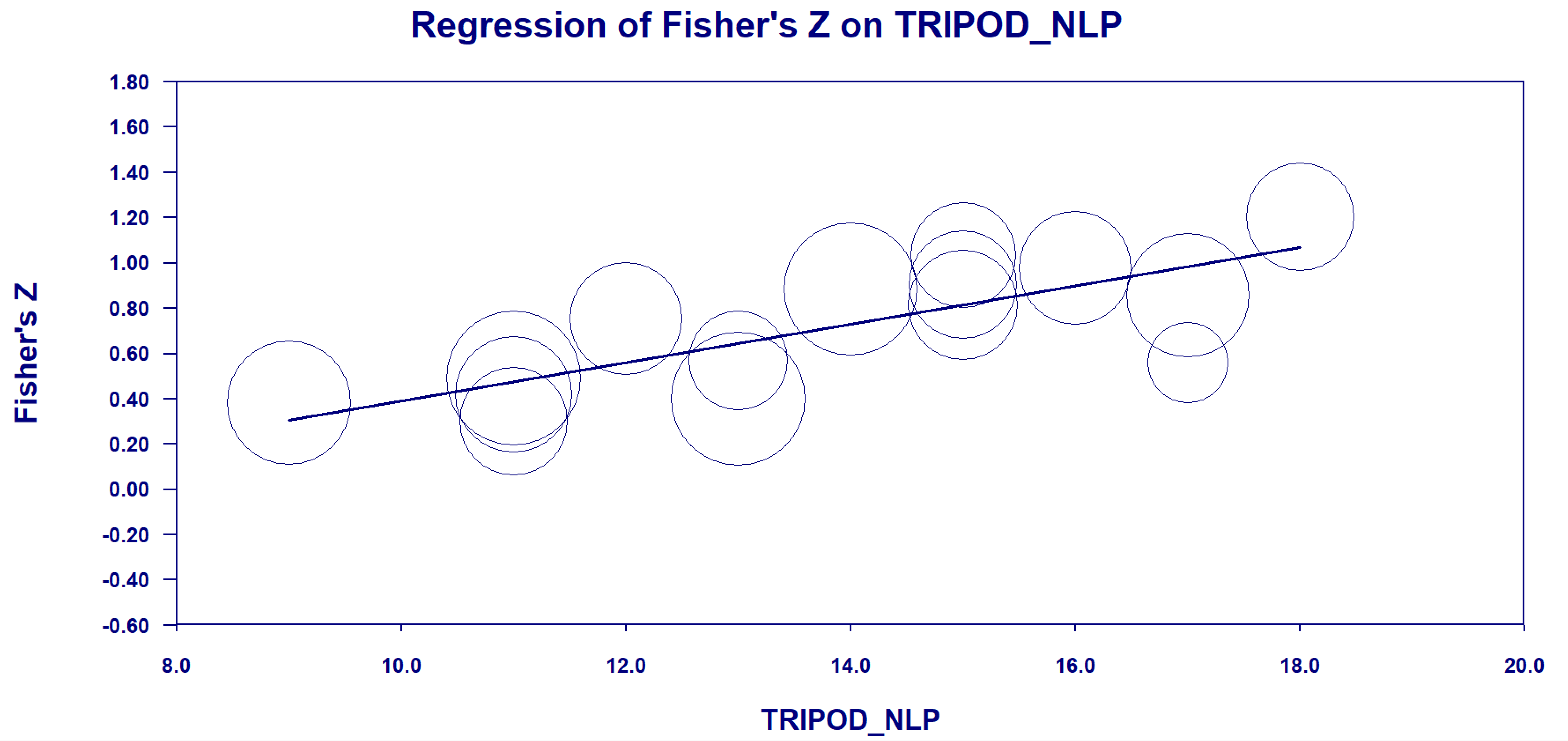


## Regression on Positive Rate


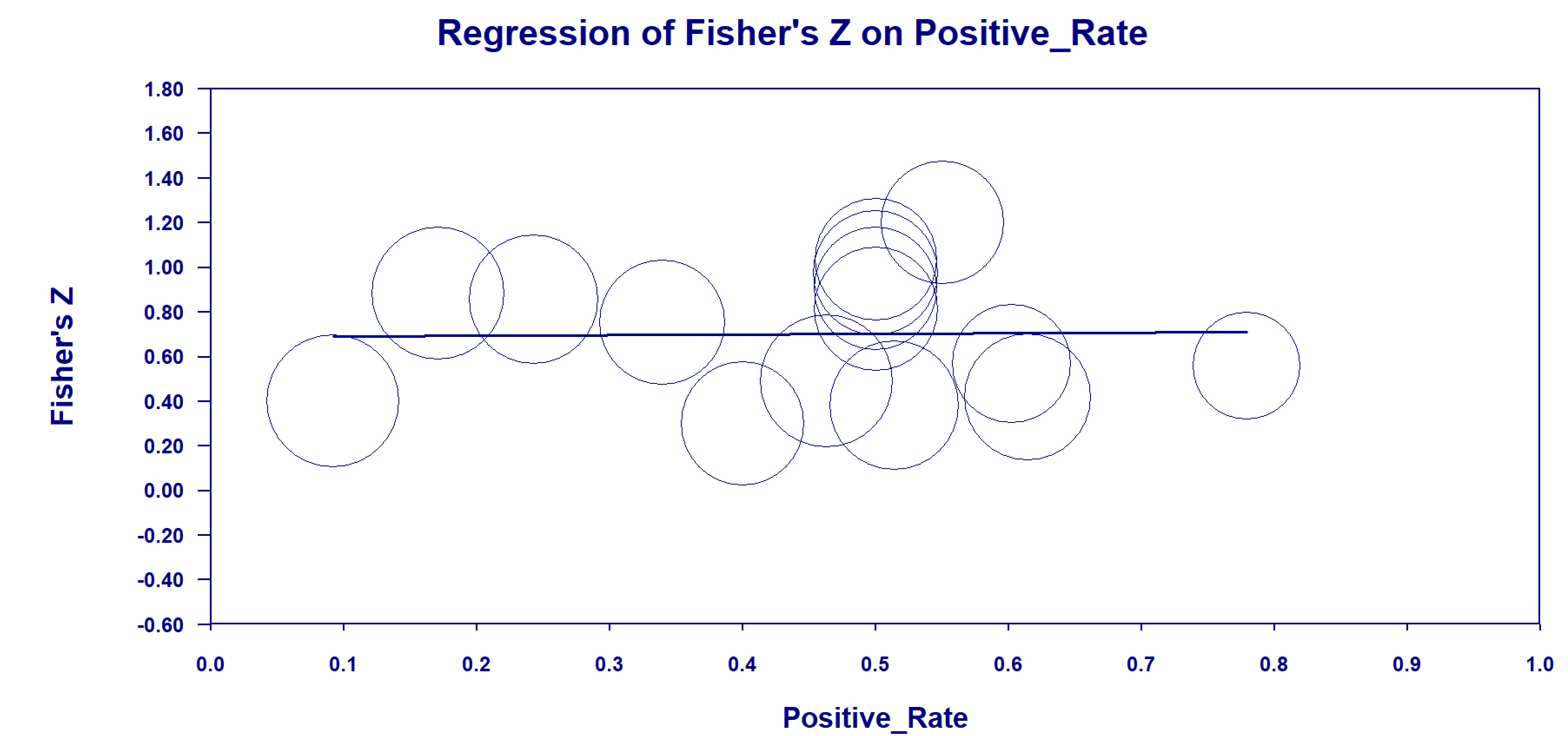


## Regression on Log-transformed Sample Size


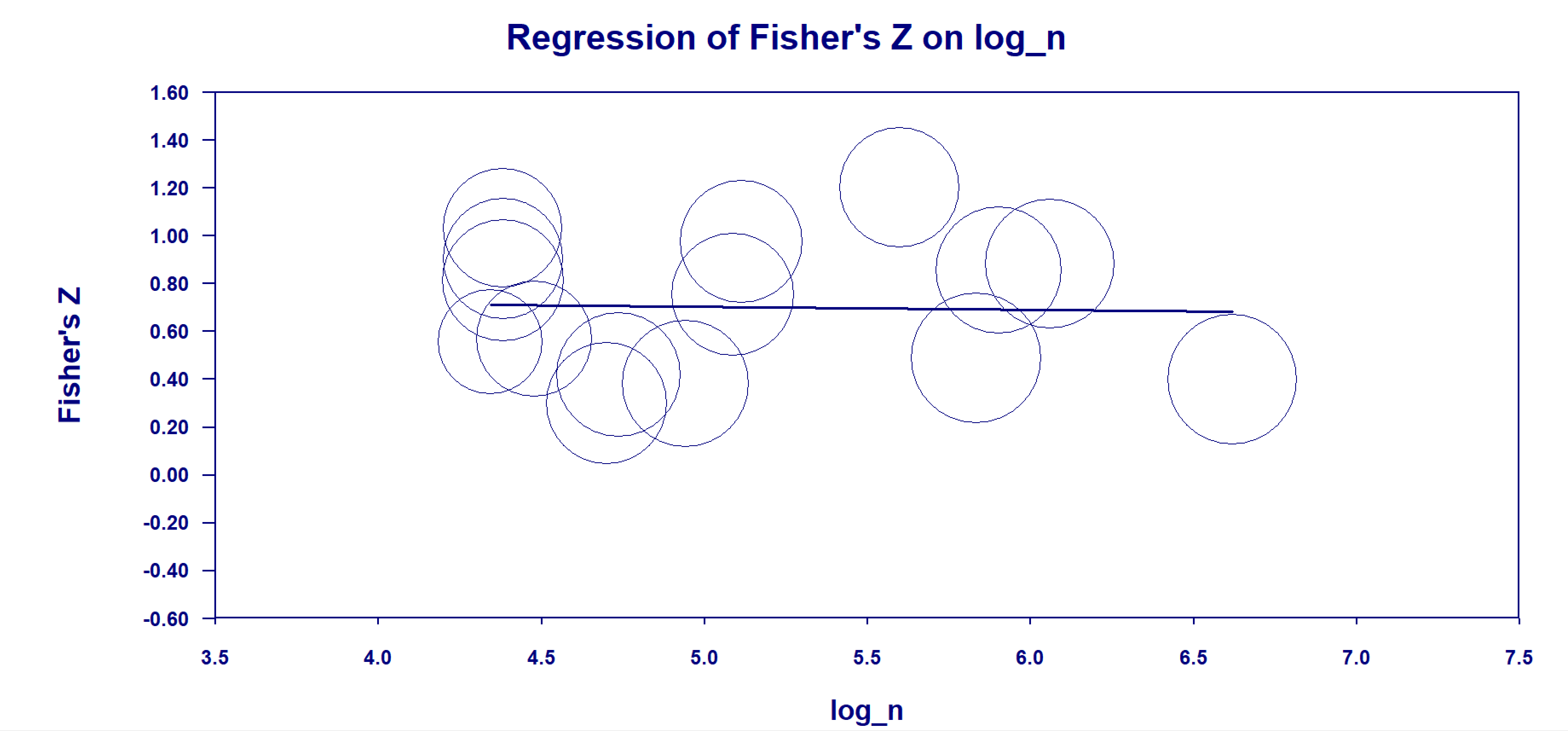

Supplement: Multimedia Appendix 6 [file jmir_v28i1e82686_app6.docx]
