# Supplementary material for: Text-Based Depression Estimation Using Machine Learning With Standard Labels: Systematic Review and Meta-Analysis
Source: J Med Internet Res. 2026 Feb 11;28:e82686. doi: 10.2196/82686 (PMC12936666; doi:10.2196/82686)
Supplement: Multimedia Appendix 7 [file jmir_v28i1e82686_app7.docx]

Supplementary Table S3. Risk of Bias Assessment for Included Studies

| Study | Participant Selection | Outcome Labeling | Text Acquisition & Preprocessing | Model Development | Reporting Transparency | Overall RoB |
| --- | --- | --- | --- | --- | --- | --- |
| Geraci et al (2017) | Low | Low | Low | Unclear | Low | Unclear |
| Ricard et al (2018) | Low | Low | Low | Low | Low | Low |
| Tlachac et al (2020) | Low | Low | Low | High | Low | High |
| Zhao et al _1 (2021) | Low | Low | Low | Unclear | Low | Unclear |
| Zhao et al _2 (2021) | Low | Low | Low | Unclear | Low | Unclear |
| Zhao et al _3 (2021) | Low | Low | Low | Unclear | Low | Unclear |
| Shin et al (2022) | Low | Low | Low | Low | Low | Low |
| Cariola et al (2022) | Low | Low | Low | Low | Low | Low |
| Munthuli et al _1 (2023) | Low | Low | Low | Low | Low | Low |
| Munthuli et al _2 (2023) | Low | Low | Low | Low | Low | Low |
| Munthuli et al _3 (2023) | Low | Low | Low | Low | Low | Low |
| Tlachac et al (2023) | Low | Low | Low | Unclear | Low | Unclear |
| Jihoon et al (2024) | Low | Low | Low | High | Low | High |
| Shin et al (2024) | Low | Low | Low | Unclear | Low | Unclear |
| Xu et al (2025) | Low | Low | Low | Low | Low | Low |

Risk of bias was evaluated across five domains tailored to text-based machine learning studies: participant selection, outcome labeling, text acquisition and preprocessing, model development, and reporting transparency. Each domain was graded as low risk, some concerns, or high risk.

Supplementary Table S4. GRADE Summary of Findings


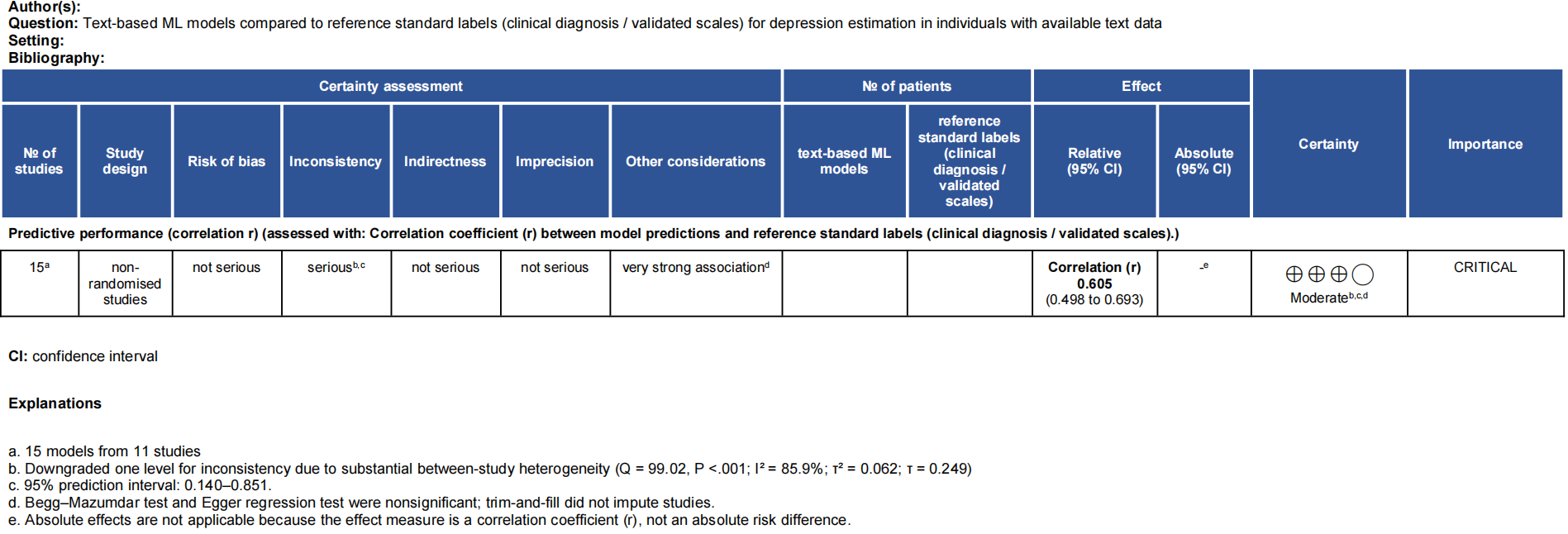


Certainty of evidence was evaluated using the GRADE framework across five domains: risk of bias, inconsistency, indirectness, imprecision, and publication bias. The outcome evaluated was the pooled predictive performance (effect size r) of models using standard depression labels.
